# Supplementary material for: Conservation prioritization can resolve the flagship species conundrum
Source: Nat Commun. 2020 Feb 24;11:994. doi: 10.1038/s41467-020-14554-z (PMC7040008; doi:10.1038/s41467-020-14554-z)
Supplement: Supplementary file 1 — Supplementary Information [file 41467_2020_14554_MOESM1_ESM.pdf]

Supplementary Information for

# Conservation prioritization can resolve the flagship species conundrum

J. McGowan[1,2,3*], L. J. Beaumont[1], R. J. Smith[4], A. L. M. Chauvenet[2,5], R. Harcourt[1], S. Atkinson[2], J. C. Mittermeier[6], M. Esperon-Rodriguez[1,7], J. B. Baumgartner[1,8], A. Beattie[1], R. Y. Dudaniec[1], R. Grenyer[6], D. A. Nipperess[1], A. Stow[1], and H. P Possingham[2,3]

1. Department of Biological Sciences, Macquarie University, NSW, 2109, Australia.
2. Centre for Biodiversity and Conservation Science, University of Queensland, QLD,4072, Australia
3. The Nature Conservancy, Arlington, VA, USA
4. Durrell Institute of Conservation and Ecology, School of Anthropology and Conservation, University of Kent, Canterbury, Kent CT2 7NR, UK
5. Environmental Futures Research Institute & School of Environment and Science, Griffith University, QLD, 4222, Australia.
6. School of Geography and Environment, Oxford University, South Parks Road, Oxford, OX1 3QY, UK.
7. Hawkesbury Institute for the Environment, Western Sydney University, 2753 NSW Australia
8. Centre of Excellence for Biosecurity Risk Analysis (CEBRA), School of BioSciences, University of Melbourne, Parkville, Victoria, Australia

Corresponding author: Jennifer McGowan, The Nature Conservancy, Arlington, Virginia, USA; email: jennifer.mcgowan@tnc.org

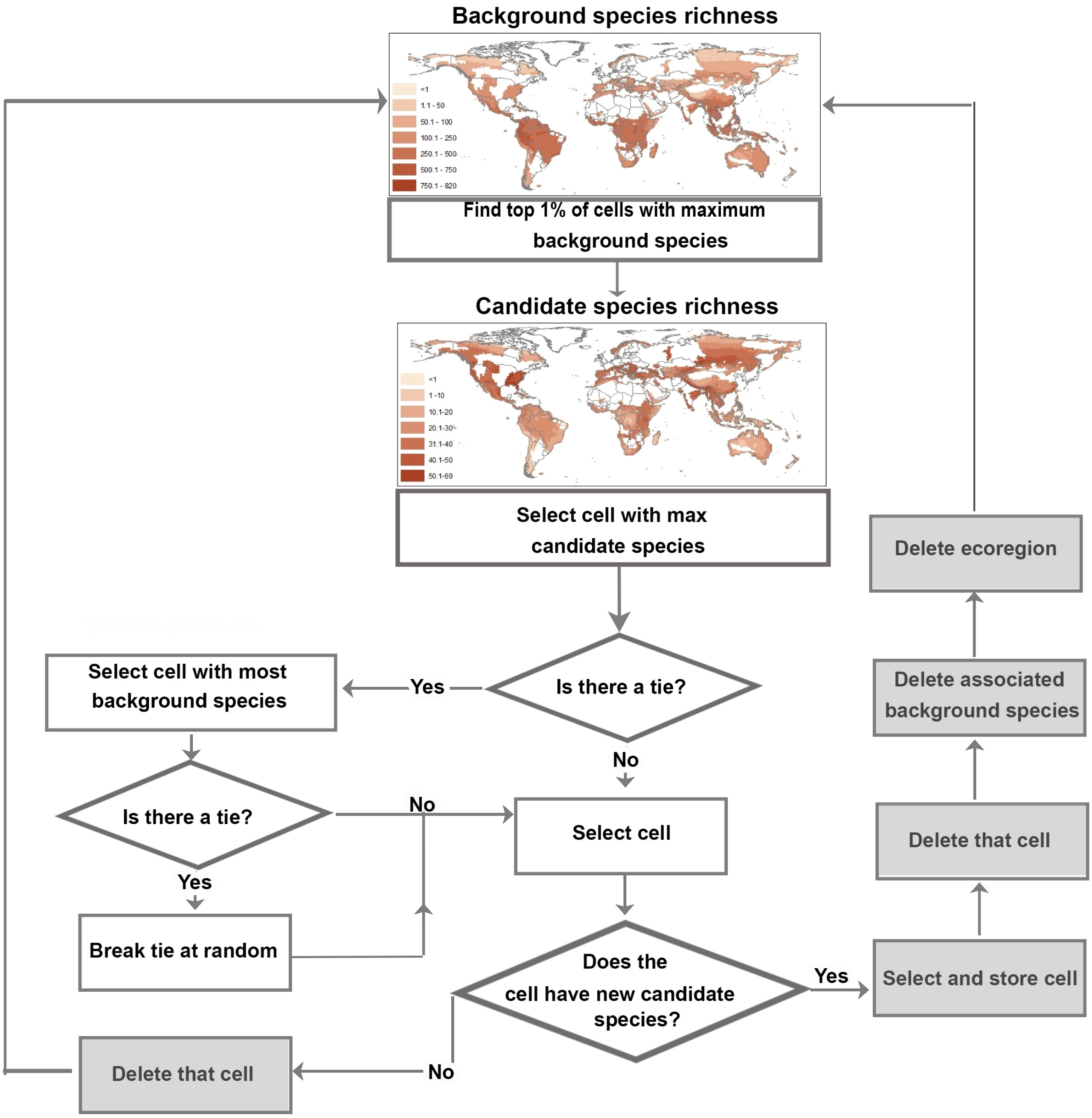


**Supplementary Figure 1. The algorithmic decision tree developed for the integrated approach**. The process begins by identifying the top 1% of sites which hold the maximum benefit (e.g. the highest numbers of background species). From that initial selection, the algorithm then picks the site which also has the most candidate flagship species. Given a tie, we preference sites with the most background species. Once a site is selected, both the ecoregion and background species associated with the site and ecoregion are deleted to ensure complementarity is achieved (cells in grey) and the algorithm cycles through the selection again until all background species have been captured. Species richness maps are from Scenario a (see Table 2 in main document).

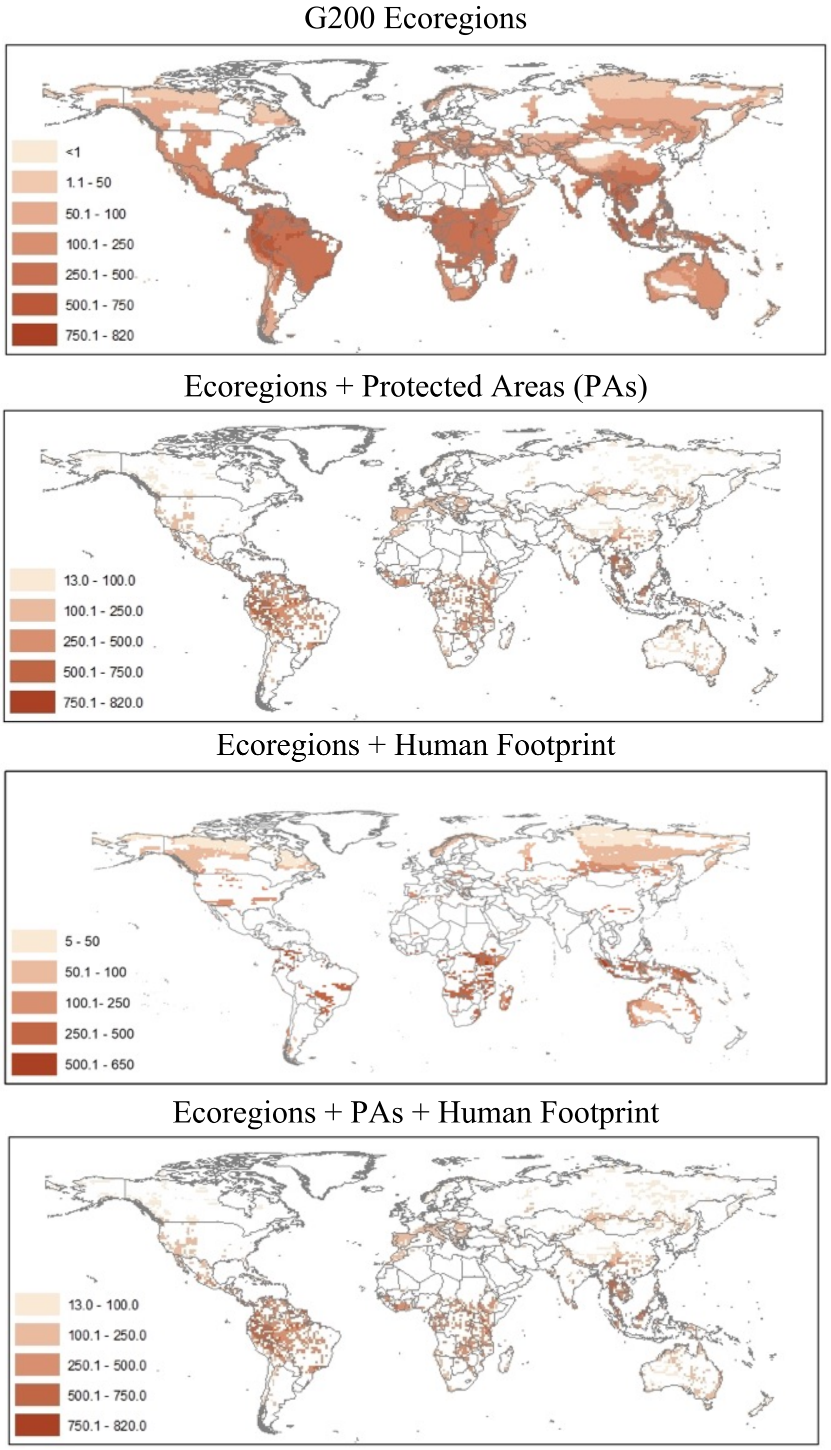


**Supplementary Figure 2. Distribution maps of the number of background species as determined by the different place-based constraints.** Note: these maps reflect the initial benefit to which we apply the algorithm from Supplementary Figure 1 and show the spatial extent of candidate places described in Table 2.

**Supplementary Methods**

We generated two randomizations of the problem. The first was a place-based randomization which was executed on Scenarios a, c, e, and g (Main document Table 2). For this analysis, we ignored the influence of flagship species, focusing solely on the accumulation of background species in the random selection for comparisons.

Additionally, we opted to conduct a second randomized test. For this, we selected a random flagship species from the candidate species list, followed by the location in its range that provided the maximum benefit. No ecoregional constraints were considered in either of the random tests and we ran 100 randomizations for each relevant scenario.

Our integrated approach outperformed both randomized tests. For the random selection of places, the mean benefit ranged from 39-55% across the suite of sites as presented in the main document (Table 1; Supplementary Figure 3). When we tested the performance of selecting a random flagship species first, followed by identifying the site in its range which delivers the maximum benefit, we found similar performance to our integrated approach (87-90% efficiency). However, these randomized analyses were not subjected to the ecoregional complementarity constraint placed on the selection of sites that our integrated approach upholds (Supplementary Figure 1). Interestingly, when we look at the top 10 most efficient sites for this species-based randomization, the average performance efficiency is 15% lower compared to our integrated approach (range 68-74%) (Supplementary Table 2, Supplementary Figure 4).

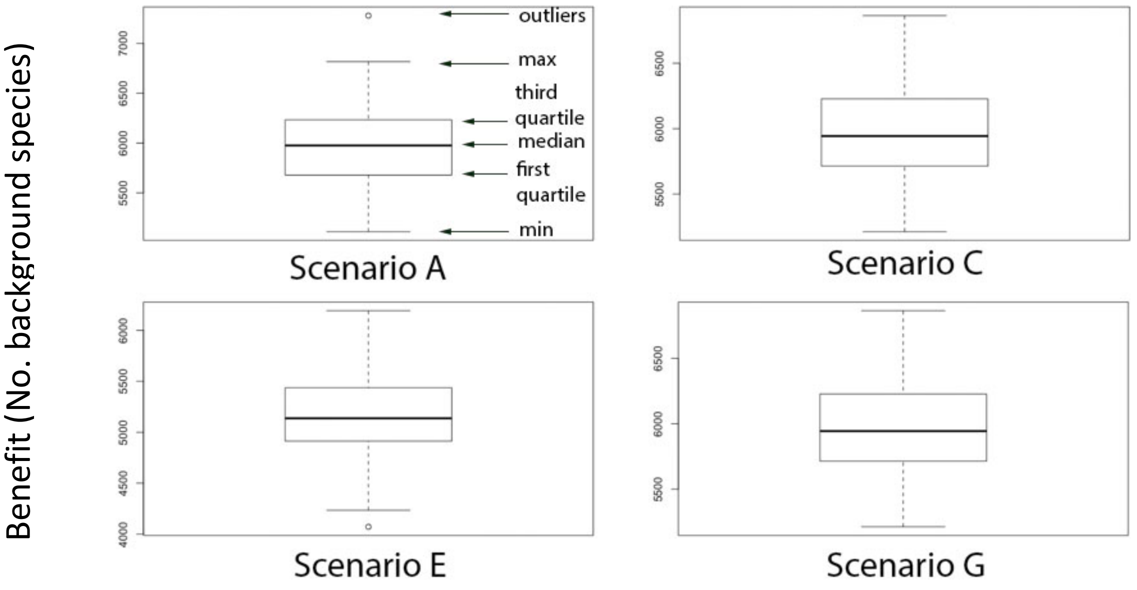


**Supplementary Figure 3. The benefit delivered from the place-based randomization test.** Boxplots show results from 100 random selections of sites for Scenario A (mean = 5,973 species); Scenario C (mean =5,968 species); Scenario E (mean = 5, 136 species); and Scenario G (mean = 5,968 species). See Source Data for raw values.

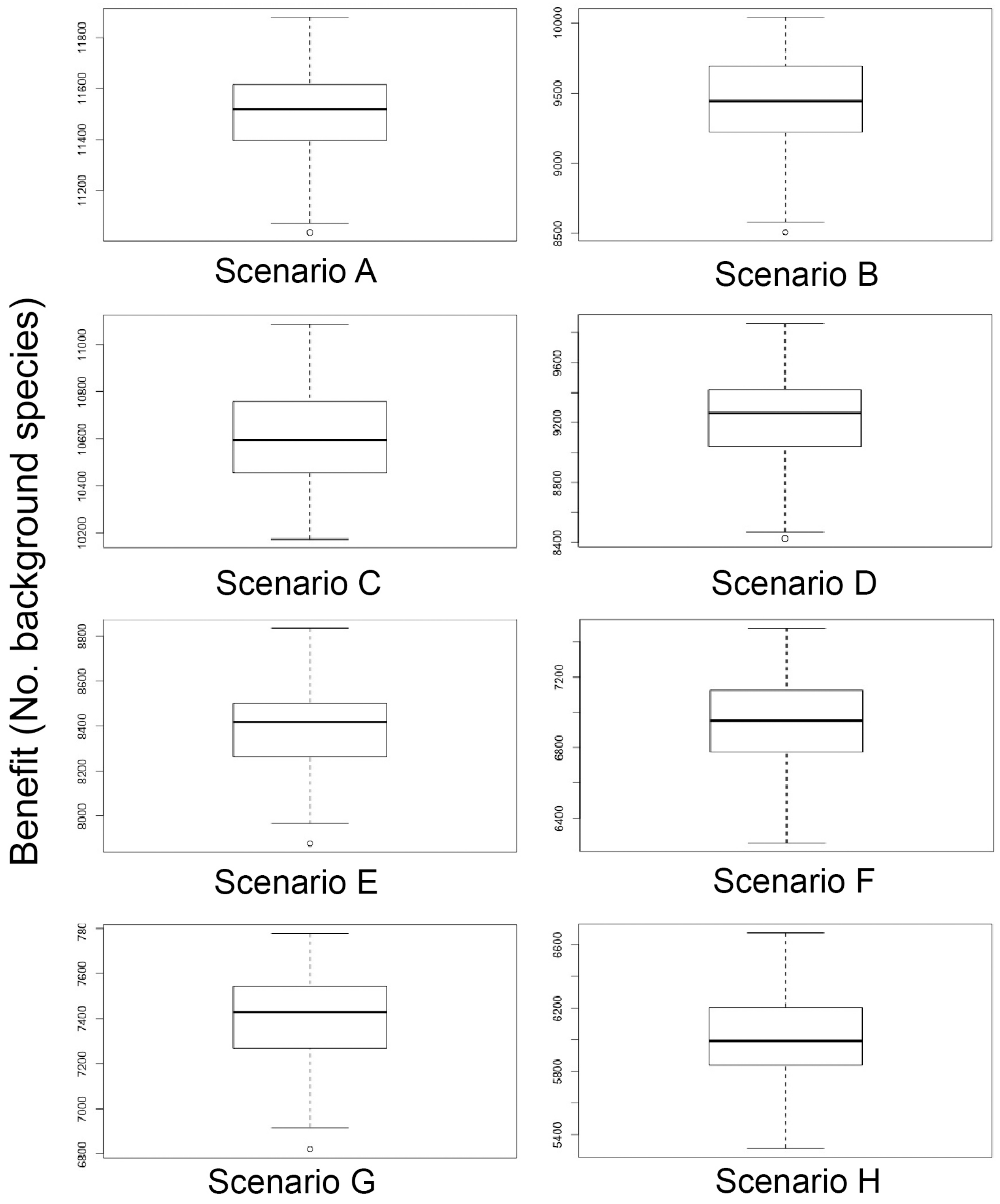


**Supplementary Figure 4. The benefit delivered from the species-based randomization test.** Boxplots show the benefit delivered from 100 random selections of species for Scenarios a-h. See Supplementary Table 2 for more details. See Source Data for values.

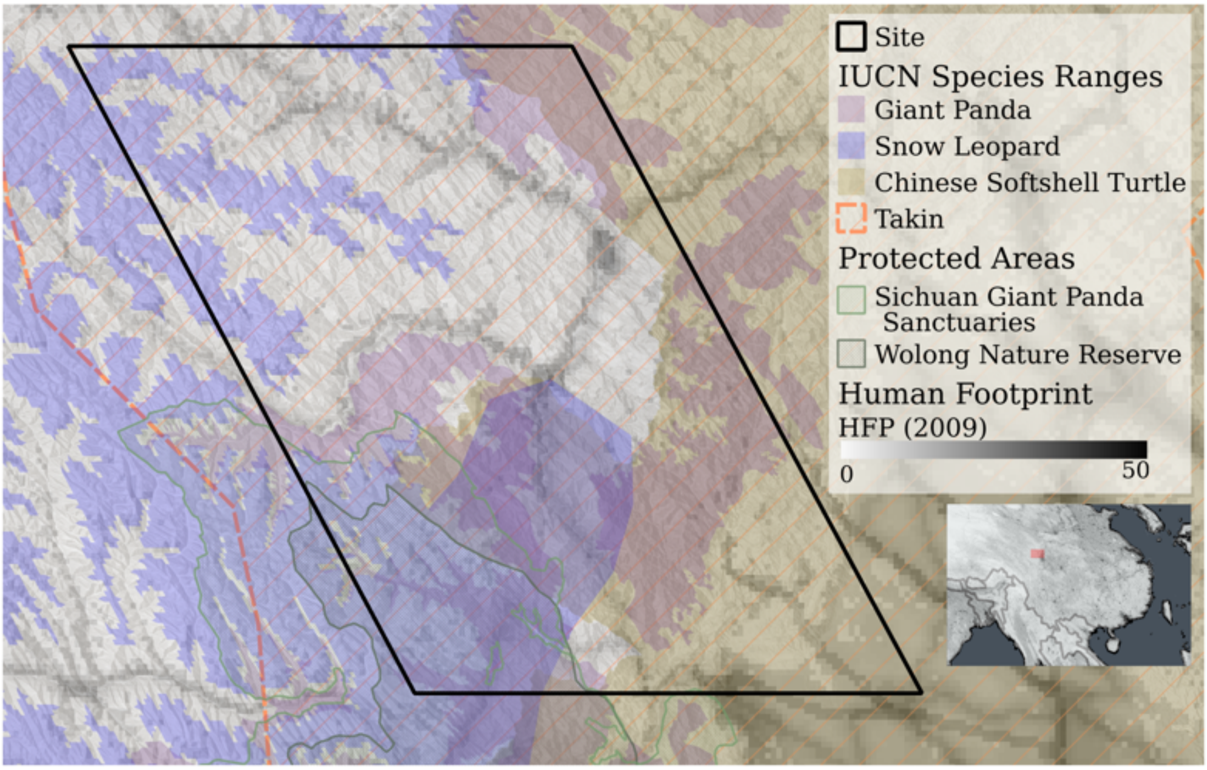


**Supplementary Figure 5**. **Example of the distribution of potential flagship species, protected areas, and human footprint within site I** (Figure 2 main document).

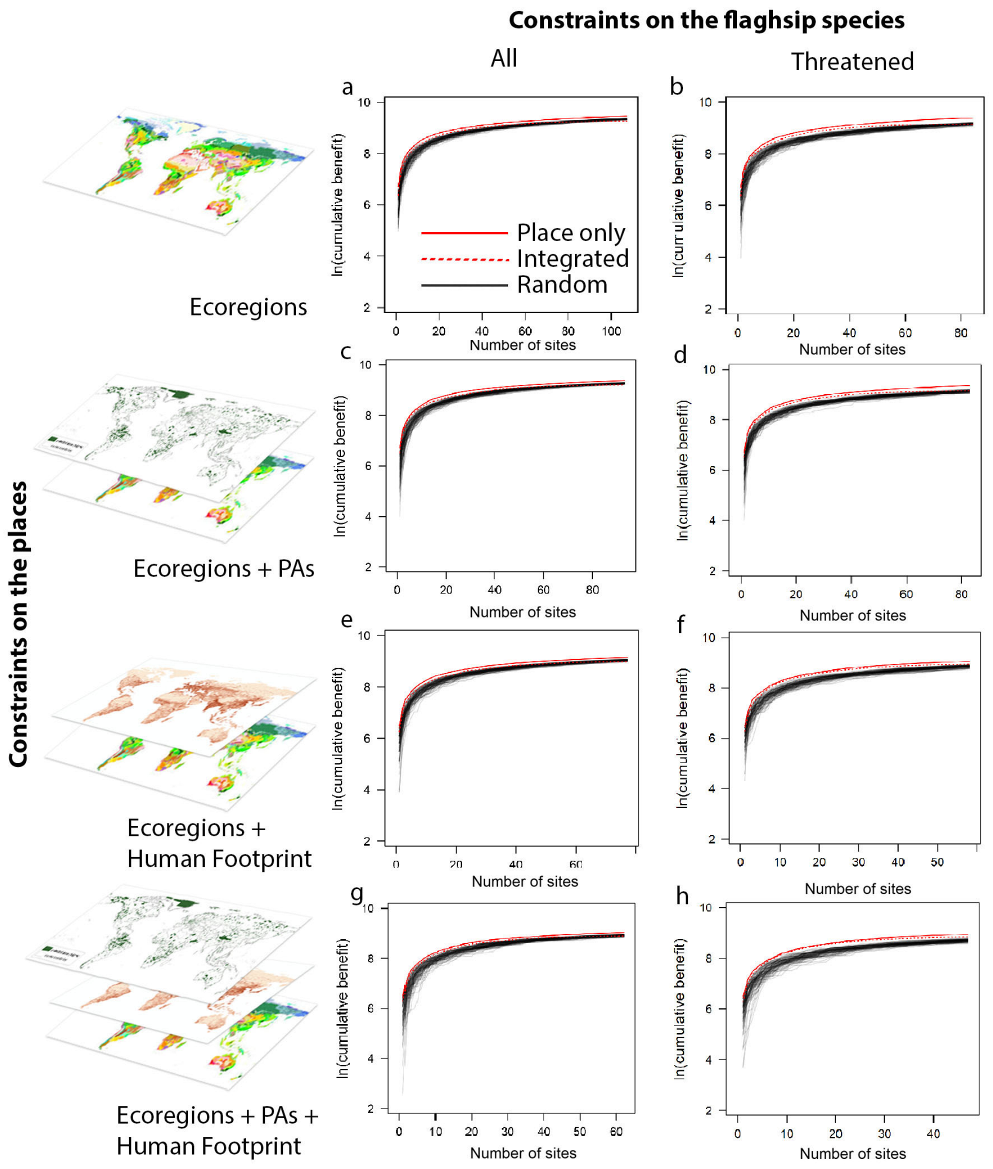


**Supplementary Figure 6. Performance comparisons across scenarios for the species-based randomized test.** The scenario performance of the place only, integrated, and random flagship species approaches (a-h) in achieving the conservation benefit (defined as the number of background species protected). Random selections were performed 100 times for each scenario. Threatened Status refers to the candidate flagship group driven by IUCN classification of Near Threatened and higher.

**Supplementary Table 1. A summary of the IUCN threat status classifications for the 534 candidate flagship species.**

| IUCN status | Candidate Birds | Candidate Mammals | Candidate Reptiles | Total |
|---|---|---|---|---|
| Critically Endangered | 12 | 62 | 4 | 78 |
| Endangered | 12 | 98 | | 110 |
| Vulnerable | 16 | 90 | 7 | 113 |
| Near Threatened | 27 | 7 | 1 | 35 |
| Least Concern | 160 | 4 | 27 | 191 |
| Lower Risk/conservation dependent | | | 1 | 1 |
| Lower Risk/least concern | | | 5 | 5 |
| Lower Risk/near threatened | | | 1 | 1 |
| Grand Total | 227 | 261 | 46 | 534 |

**Supplementary Table 2. Results from the place-only, integrated, and random species approaches**. Values describe the maximum benefit available for each scenario (Scenarios a-h) as described in Table 2 and Supplementary Figure 6. We report on the mean benefit of 100 prioritizations for the randomized tests.

| | Max benefit (No. of background species) | No. sites in solution: | Number of species delivered with: | | | Percent of efficiency retained for full suite of sites: | | Percent of efficiency retained for top 10 sites: | |
|---|---|---|---|---|---|---|---|---|---|
| Scenario | | integrated approach | place-only approach | integrated approach | Species-based random test | integrated | Species-based null (mean 100 runs) | integrated | Species-based random test |
| a | 19,616 | 107 | 12,878 | 10,545 | 11,493 | 82% | 89% | 87% | 72% |
| b | 19,616 | 84 | 11,961 | 9,487 | 9,448 | 79% | 79% | 90% | 68% |
| c | 16,542 | 93 | 11,835 | 9,965 | 10,596 | 84% | 90% | 92% | 74% |
| d | 16,542 | 83 | 11,443 | 9,387 | 9,220 | 82% | 81% | 92% | 69% |
| e | 12,053 | 77 | 9,362 | 7,972 | 8,391 | 85% | 90% | 89% | 74% |
| f | 12,053 | 58 | 8,557 | 7,621 | 6,935 | 89% | 81% | 96% | 71% |
| g | 9,833 | 62 | 8,363 | 7,269 | 7,400 | 87% | 88% | 93% | 74% |
| h | 9,833 | 47 | 7,702 | 6,849 | 6,017 | 89% | 78% | 97% | 71% |

**Supplementary Table 3. The locations of the 47 sites associated with Fig 2 and Supplementary Data Table 2.**

| Site_ID | Ecoregion | Country |
|---|---|---|
| 1 | Sumatran Islands Lowland and Montane Forests | Indonesia |
| 2 | East African Acacia Savannas | Kenya |
| 3 | Rio Negro-Jurua Moist Forests | Peru |
| 4 | Guinean Moist Forests | Cote d'Ivoire/Liberia |
| 5 | Northern Andean Montane Forests | Ecuador |
| 6 | Naga-Manapuri-Chin Hills Moist Forests | India/Bhutan |
| 7 | Madagascar Forests and Shrublands | Madagascar |
| 8 | Mesoamerican Pine-Oak Forests | Guatemala |
| 9 | New Guinea Montane Forests | Papua New Guinea |
| 10 | Choco-Darien Moist Forests | Colombia |
| 11 | East African Coastal Forests | United Republic of Tanzania |
| 12 | Cerrado Woodlands and Savannas | Brazil |
| 13 | Congolian Coastal Forests | Gabon/Equatorial Guinea |
| 14 | Appalachian and Mixed Mesophytic Forests | United States |
| 15 | Chihuahuan-Tehuacan Deserts | Mexico/United States |
| 16 | Hengduan Shan Conifer Forests | China |
| 17 | Philippines Moist Forests | Philippines |
| 18 | Madagascar Spiny Thicket | Madagascar |
| 20 | Borneo Lowland and Montane Forests | Indonesia/Malaysia |
| 25 | Drakensberg Montane Woodlands and Grasslands | South Africa |
| 26 | Sulawesi Moist Forests | Indonesia |
| 28 | European-Mediterranean Montane Forests | Greece/Bulgaria |
| 34 | Amazon River and Flooded Forests | Brazil |
| 35 | Albertine Rift Montane Forests | Uganda/Democratic Republic of the Congo |
| 43 | Central and Eastern Miombo Woodlands | Angola/Zambia |
| 52 | Horn of Africa Acacia Savannas | Ethiopia |
| 63 | Mediterranean Forests, Woodlands and Scrub | Algeria |
| 82 | Valdivian Temperate Rain Forests / Juan Fern□«□□nde | Chile |
| 95 | Atlantic Dry Forests | Brazil |
| 101 | Guianan Highlands Moist Forests | Venezuela |
| 102 | Altai-Sayan Montane Forests | Kazakhstan |
| 106 | Madagascar Dry Forests | Madagascar |
| 112 | Lower Mississippi River | United States |
| 124 | Eastern Arc Montane Forests | United Republic of Tanzania |
| 129 | Russian Far East Broadleaf and Mixed Forests | China/Russia |
| 130 | Sonoran-Baja Deserts | Mexico |

| | | |
|---|---|---|
| 131 | Southwestern Amazonian Moist Forests | Bolivia |
| 137 | Colorado River | United States |
| 145 | Moluccas Moist Forests | Indonesia |
| 146 | Namib-Karoo-Kaokoveld Deserts and Shrublands | Namibia |
| 152 | New Zealand Temperate Forests | New Zealand |
| 157 | Chihuahuan Freshwater | Mexico |
| 159 | Patagonian Steppe | Argentina |
| 161 | New Guinea Rivers & Streams | Indonesia |
| 403 | Northern Prairies | Canada |
| 527 | Volga River Delta | Russia |
| 589 | Greater Sundas Mangroves | Indonesia |
