# Supplementary material for: Conservation prioritization can resolve the flagship species conundrum
Source: Nat Commun. 2020 Feb 24;11:994. doi: 10.1038/s41467-020-14554-z (PMC7040008; doi:10.1038/s41467-020-14554-z)
Supplement: Supplementary file 3 — Description of Additional Supplementary Information [file 41467_2020_14554_MOESM3_ESM.docx]

**Description of Additional Supplementary Files**

**File Name:** Supplementary Data Table 1
**Description:** Full list of candidate flagships. Scientific and common name, threat status of 534 candidate flagship species consisting of 227 birds, 261 mammals, and 46 reptiles.

**File Name:** Supplementary Data Table 2
**Description:** List of flagship species resulting from the integrated approach for scenario h (Table 2 in main document).
