# Supplementary material for: Conservation prioritization can resolve the flagship species conundrum
Source: Nat Commun. 2020 Feb 24;11:994. doi: 10.1038/s41467-020-14554-z (PMC7040008; doi:10.1038/s41467-020-14554-z)
Supplement: Supplementary file 4 — Supplementary Data Table 1 [file 41467_2020_14554_MOESM4_ESM.docx]

Supplementary Data for

**Conservation prioritization can resolve the flagship species conundrum**

J. McGowan^1,2,3*^, L. J. Beaumont^1^, R. J. Smith^4^, A. L. M. Chauvenet^2,5^, R. Harcourt^1^, S. Atkinson^2^, J. C. Mittermeier^6^, M. Esperon-Rodriguez^1,7^, J. B. Baumgartner^1,8^, A. Beattie^1^, R. Y. Dudaniec^1^, R. Grenyer^6^, D. A. Nipperess^1^, A. Stow^1^, and H. P Possingham^2,3^

**Supplementary Data Table 1.** **Full list of candidate flagships.** Scientific and common name, threat status of 534 candidate flagship species consisting of 227 birds, 261 mammals, and 46 reptiles.

| **Scientific name** | **Common name** | **Threat status** | **Type** |
| --- | --- | --- | --- |
| *Accipiter badius* | Shikra | Least Concern | Candidate Bird |
| *Accipiter cooperii* | Cooper's Hawk | Least Concern | Candidate Bird |
| *Accipiter gentilis* | Northern Goshawk | Least Concern | Candidate Bird |
| *Accipiter nisus* | Eurasian Sparrowhawk | Least Concern | Candidate Bird |
| *Aegolius acadicus* | Northern Saw-whet Owl | Least Concern | Candidate Bird |
| *Aegolius funereus* | Boreal Owl | Least Concern | Candidate Bird |
| *Aegypius monachus* | Cinereous vulture | Near Threatened | Candidate Bird |
| *Afropavo congensis* | Congo Peafowl | Vulnerable | Candidate Bird |
| *Agapornis fischeri* | Fischer's lovebird | Near Threatened | Candidate Bird |
| *Agapornis roseicollis* | Rosy-faced Lovebird | Least Concern | Candidate Bird |
| *Aix galericulata* | Mandarin Duck | Least Concern | Candidate Bird |
| *Aix sponsa* | Wood Duck | Least Concern | Candidate Bird |
| *Alcedo atthis* | Common Kingfisher | Least Concern | Candidate Bird |
| *Alectoris chukar* | Chukar | Least Concern | Candidate Bird |
| *Alectura lathami* | Australian Brush-turkey | Least Concern | Candidate Bird |
| *Alisterus scapularis* | Australian King-parrot | Least Concern | Candidate Bird |
| *Alopochen aegyptiaca* | Egyptian Goose | Least Concern | Candidate Bird |
| *Amazona oratrix* | Yellow-headed amazon | Endangered | Candidate Bird |
| *Anas acuta* | Northern Pintail | Least Concern | Candidate Bird |
| *Anas platyrhynchos* | Mallard | Least Concern | Candidate Bird |
| *Anas rubripes* | American Black Duck | Least Concern | Candidate Bird |
| *Anodorhynchus hyacinthinus* | Hyacinth macaw | Vulnerable | Candidate Bird |
| *Anser albifrons* | Greater White-fronted Goose | Least Concern | Candidate Bird |
| *Anser anser* | Greylag Goose | Least Concern | Candidate Bird |
| *Anser caerulescens* | Snow Goose | Least Concern | Candidate Bird |
| *Anser indicus* | Bar-headed Goose | Least Concern | Candidate Bird |
| *Anthropoides paradiseus* | Blue crane | Vulnerable | Candidate Bird |
| *Anthropoides virgo* | Demoiselle Crane | Least Concern | Candidate Bird |
| *Apus apus* | Common Swift | Least Concern | Candidate Bird |
| *Aquila audax* | Wedge-tailed Eagle | Least Concern | Candidate Bird |
| *Aquila chrysaetos* | Golden Eagle | Least Concern | Candidate Bird |
| *Aquila heliaca* | Eastern imperial eagle | Vulnerable | Candidate Bird |
| *Aquila nipalensis* | Steppe Eagle | Endangered | Candidate Bird |
| *Aquila rapax* | Tawny Eagle | Least Concern | Candidate Bird |
| *Ara ararauna* | Blue-and-yellow Macaw | Least Concern | Candidate Bird |
| *Ara chloropterus* | Red-and-green Macaw | Least Concern | Candidate Bird |
| *Ara glaucogularis* | Blue-throated macaw | Critically Endangered | Candidate Bird |
| *Ara macao* | Scarlet Macaw | Least Concern | Candidate Bird |
| *Ara militaris* | Military macaw | Vulnerable | Candidate Bird |
| *Archilochus colubris* | Ruby-throated Hummingbird | Least Concern | Candidate Bird |
| *Ardeotis kori* | Kori bustard | Near Threatened | Candidate Bird |
| *Ardeotis nigriceps* | Great Indian Bustard | Critically Endangered | Candidate Bird |
| *Asio flammeus* | Short-eared Owl | Least Concern | Candidate Bird |
| *Asio otus* | Northern Long-eared Owl | Least Concern | Candidate Bird |
| *Athene cunicularia* | Burrowing Owl | Least Concern | Candidate Bird |
| *Athene noctua* | Little Owl | Least Concern | Candidate Bird |
| *Aythya fuligula* | Tufted Duck | Least Concern | Candidate Bird |
| *Aythya marila* | Greater Scaup | Least Concern | Candidate Bird |
| *Aythya valisineria* | Canvasback | Least Concern | Candidate Bird |
| *Balearica regulorum* | Grey Crowned-crane | Endangered | Candidate Bird |
| *Barnardius zonarius* | Australian Ringneck | Least Concern | Candidate Bird |
| *Bonasa umbellus* | Ruffed Grouse | Least Concern | Candidate Bird |
| *Branta canadensis* | Canada Goose | Least Concern | Candidate Bird |
| *Branta leucopsis* | Barnacle Goose | Least Concern | Candidate Bird |
| *Bubo bengalensis* | Rock Eagle-owl | Least Concern | Candidate Bird |
| *Bubo blakistoni* | Blakiston'sfish owl | Endangered | Candidate Bird |
| *Bubo bubo* | Eurasian Eagle-owl | Least Concern | Candidate Bird |
| *Bubo scandiacus* | Snowy Owl | Vulnerable | Candidate Bird |
| *Bucephala albeola* | Bufflehead | Least Concern | Candidate Bird |
| *Bucephala clangula* | Common Goldeneye | Least Concern | Candidate Bird |
| *Buceros bicornis* | Great Hornbill | Near Threatened | Candidate Bird |
| *Buceros rhinoceros* | Rhonocerous hornbill | Near Threatened | Candidate Bird |
| *Burhinus grallarius* | Bush Thick-knee | Least Concern | Candidate Bird |
| *Buteo jamaicensis* | Red-tailed Hawk | Least Concern | Candidate Bird |
| *Buteo lagopus* | Rough-legged Buzzard | Least Concern | Candidate Bird |
| *Buteo lineatus* | Red-shouldered Hawk | Least Concern | Candidate Bird |
| *Buteo platypterus* | Broad-winged Hawk | Least Concern | Candidate Bird |
| *Buteo regalis* | Ferruginous Hawk | Least Concern | Candidate Bird |
| *Buteo swainsoni* | Swainson's Hawk | Least Concern | Candidate Bird |
| *Cacatua alba* | White Cockatoo | Endangered | Candidate Bird |
| *Cacatua galerita* | Sulphur-crested Cockatoo | Least Concern | Candidate Bird |
| *Cacatua leadbeateri* | Major Mitchell's Cockatoo | Least Concern | Candidate Bird |
| *Cacatua moluccensis* | Salmon-crested Cockatoo | Vulnerable | Candidate Bird |
| *Cacatua sulphurea* | Yellow-crested cockatoo | Critically Endangered | Candidate Bird |
| *Cairina moschata* | Muscovy Duck | Least Concern | Candidate Bird |
| *Calidris alba* | Sanderling | Least Concern | Candidate Bird |
| *Calidris canutus* | Red Knot | Near Threatened | Candidate Bird |
| *Callipepla californica* | California Quail | Least Concern | Candidate Bird |
| *Callipepla gambelii* | Gambel's Quail | Least Concern | Candidate Bird |
| *Caloenas nicobarica* | Nicobar pigeon | Near Threatened | Candidate Bird |
| *Calypte anna* | Anna's Hummingbird | Least Concern | Candidate Bird |
| *Calyptorhynchus banksii* | Red-tailed Black-cockatoo | Least Concern | Candidate Bird |
| *Campephilus principalis* | Ivory-billed woodpecker | Critically Endangered | Candidate Bird |
| *Caracara cheriway* | Crested Caracara | Least Concern | Candidate Bird |
| *Casuarius bennetti* | Dwarf cassowary | Least Concern | Candidate Bird |
| *Casuarius casuarius* | Southern Cassowary | Least Concern | Candidate Bird |
| *Casuarius unappendiculatus* | Northern Cassowary | Least Concern | Candidate Bird |
| *Centrocercus urophasianus* | Greater sage-grouse | Near Threatened | Candidate Bird |
| *Centropus sinensis* | Greater Coucal | Least Concern | Candidate Bird |
| *Chaetura pelagica* | Chimney swift | Near Threatened | Candidate Bird |
| *Charadrius melodus* | Piping plover | Near Threatened | Candidate Bird |
| *Charadrius vociferus* | Killdeer | Least Concern | Candidate Bird |
| *Chordeiles minor* | Common Nighthawk | Least Concern | Candidate Bird |
| *Clangula hyemalis* | Long-tailed Duck | Vulnerable | Candidate Bird |
| *Columba livia* | Rock Dove | Least Concern | Candidate Bird |
| *Columba palumbus* | Common Woodpigeon | Least Concern | Candidate Bird |
| *Coracias caudatus* | Lilac-breasted Roller | Least Concern | Candidate Bird |
| *Coturnix coturnix* | Common Quail | Least Concern | Candidate Bird |
| *Coturnix japonica* | Japanese quail | Near Threatened | Candidate Bird |
| *Crex crex* | Corncrake | Least Concern | Candidate Bird |
| *Cuculus canorus* | Common Cuckoo | Least Concern | Candidate Bird |
| *Cyanopsitta spixii* | Spix's Macaw | Critically Endangered | Candidate Bird |
| *Cygnus atratus* | Black Swan | Least Concern | Candidate Bird |
| *Cygnus buccinator* | Trumpeter Swan | Least Concern | Candidate Bird |
| *Cygnus columbianus* | Tundra Swa | Least Concern | Candidate Bird |
| *Cygnus cygnus* | Whooper Swan | Least Concern | Candidate Bird |
| *Cygnus olor* | Mute Swan | Least Concern | Candidate Bird |
| *Dacelo novaeguineae* | Laughing Kookaburra | Least Concern | Candidate Bird |
| *Dendrocopos major* | Great Spotted Woodpecker | Least Concern | Candidate Bird |
| *Dendrocygna autumnalis* | Black-bellied Whistling-duck | Least Concern | Candidate Bird |
| *Dendrocygna bicolor* | Fulvous Whistling-duck | Least Concern | Candidate Bird |
| *Dromaius novaehollandiae* | Common Emu | Least Concern | Candidate Bird |
| *Dryobates pubescens* | Downy Woodpecker | Least Concern | Candidate Bird |
| *Eclectus roratus* | Eclectus Parrot | Least Concern | Candidate Bird |
| *Elanoides forficatus* | Swallow-tailed Kite | Least Concern | Candidate Bird |
| *Elanus leucurus* | White-tailed Kite | Least Concern | Candidate Bird |
| *Eolophus roseicapilla* | Galah | Least Concern | Candidate Bird |
| *Eudynamys scolopaceus* | Western Koel | Least Concern | Candidate Bird |
| *Falco amurensis* | Amur Falcon | Least Concern | Candidate Bird |
| *Falco columbarius* | Merlin | Least Concern | Candidate Bird |
| *Falco sparverius* | American Kestrel | Least Concern | Candidate Bird |
| *Falco tinnunculus* | Common Kestrel | Least Concern | Candidate Bird |
| *Francolinus francolinus* | Black Francolin | Least Concern | Candidate Bird |
| *Francolinus pondicerianus* | Grey Francolin | Least Concern | Candidate Bird |
| *Fulica atra* | Common Coot | Least Concern | Candidate Bird |
| *Gallinago gallinago* | Common Snipe | Least Concern | Candidate Bird |
| *Gallirallus australis* | Weka | Vulnerable | Candidate Bird |
| *Gallus gallus* | Red Junglefowl | Least Concern | Candidate Bird |
| *Gallus sonneratii* | Grey Junglefowl | Least Concern | Candidate Bird |
| *Geococcyx californianus* | Greater Roadrunner | Least Concern | Candidate Bird |
| *Grus americana* | Whooping Crane | Endangered | Candidate Bird |
| *Grus grus* | Common Crane | Least Concern | Candidate Bird |
| *Grus japonensis* | Red-crowned Crane | Endangered | Candidate Bird |
| *Grus nigricollis* | Black-necked Crane | Vulnerable | Candidate Bird |
| *Gypaetus barbatus* | Bearded vulture | Near Threatened | Candidate Bird |
| *Gyps africanus* | White backed vulture | Critically Endangered | Candidate Bird |
| *Gyps bengalensis* | White-rumped vulture | Critically Endangered | Candidate Bird |
| *Gyps fulvus* | Griffon Vulture | Least Concern | Candidate Bird |
| *Haliaeetus albicilla* | White-tailed Sea-eagle | Least Concern | Candidate Bird |
| *Haliaeetus leucocephalus* | Bald Eagle | Least Concern | Candidate Bird |
| *Haliaeetus leucogaster* | White-bellied Sea-eagle | Least Concern | Candidate Bird |
| *Haliaeetus pelagicus* | Steller's Sea-eagle | Vulnerable | Candidate Bird |
| *Haliaeetus vocifer* | African Fish-eagle | Least Concern | Candidate Bird |
| *Haliastur indus* | Brahminy Kite | Least Concern | Candidate Bird |
| *Harpia harpyja* | Harpy eagle | Near Threatened | Candidate Bird |
| *Heteroglaux blewitti* | Forest Owlet | Endangered | Candidate Bird |
| *Hieraaetus pennatus* | Booted Eagle | Least Concern | Candidate Bird |
| *Hirundapus caudacutus* | White-throated Needletail | Least Concern | Candidate Bird |
| *Histrionicus histrionicus* | Harlequin Duck | Least Concern | Candidate Bird |
| *Ictinia mississippiensis* | Mississippi Kite | Least Concern | Candidate Bird |
| *Lagopus lagopus* | Willow Grouse | Least Concern | Candidate Bird |
| *Lagopus muta* | Rock Ptarmigan | Least Concern | Candidate Bird |
| *Leuconotopicus borealis* | Red-cockaded woodpecker | Near Threatened | Candidate Bird |
| *Leuconotopicus villosus* | Hairy Woodpecker | Least Concern | Candidate Bird |
| *Limosa lapponica* | Bar-tailed Godwit | Near Threatened | Candidate Bird |
| *Lophodytes cucullatus* | Hooded Merganser | Least Concern | Candidate Bird |
| *Lophophorus impejanus* | Himalayan Monal | Least Concern | Candidate Bird |
| *Lyrurus tetrix* | Black Grouse | Least Concern | Candidate Bird |
| *Megaceryle alcyon* | Belted Kingfisher | Least Concern | Candidate Bird |
| *Megascops asio* | Eastern Screech-owl | Least Concern | Candidate Bird |
| *Melanerpes carolinus* | Red-bellied Woodpecker | Least Concern | Candidate Bird |
| *Melanerpes erythrocephalus* | Red-headed woodpecker | Near Threatened | Candidate Bird |
| *Melanerpes formicivorus* | Acorn Woodpecker | Least Concern | Candidate Bird |
| *Meleagris gallopavo* | Wild Turkey | Least Concern | Candidate Bird |
| *Meleagris ocellata* | Ocellated Turkey | Near Threatened | Candidate Bird |
| *Mellisuga helenae* | Bee Hummingbird | Near Threatened | Candidate Bird |
| *Melopsittacus undulatus* | Budgerigar | Least Concern | Candidate Bird |
| *Mergellus albellus* | Smew | Least Concern | Candidate Bird |
| *Mergus merganser* | Goosander | Least Concern | Candidate Bird |
| *Mergus serrator* | Red-breasted Merganser | Least Concern | Candidate Bird |
| *Merops apiaster* | European Bee-eater | Least Concern | Candidate Bird |
| *Micrathene whitneyi* | Elf Owl | Least Concern | Candidate Bird |
| *Milvus milvus* | Red kite | Near Threatened | Candidate Bird |
| *Neophron percnopterus* | Egyptian Vulture | Endangered | Candidate Bird |
| *Nestor notabilis* | Kea | Endangered | Candidate Bird |
| *Ninox strenua* | Powerful Owl | Least Concern | Candidate Bird |
| *Numenius borealis* | Eskimo Curlew | Critically Endangered | Candidate Bird |
| *Numida meleagris* | Helmeted Guineafowl | Least Concern | Candidate Bird |
| *Nyctibius grandis* | Great Potoo | Least Concern | Candidate Bird |
| *Nymphicus hollandicus* | Cockatiel | Least Concern | Candidate Bird |
| *Opisthocomus hoazin* | Hoatzin | Least Concern | Candidate Bird |
| *Otis tarda* | Great Bustard | Vulnerable | Candidate Bird |
| *Pandion haliaetus* | Osprey | Least Concern | Candidate Bird |
| *Parabuteo unicinctus* | Harris's Hawk | Least Concern | Candidate Bird |
| *Pavo cristatus* | Indian Peafowl | Least Concern | Candidate Bird |
| *Pavo muticus* | Green Peafowl | Endangered | Candidate Bird |
| *Perdix perdix* | Grey Partridge | Least Concern | Candidate Bird |
| *Pernis apivorus* | European Honey-buzzard | Least Concern | Candidate Bird |
| *Pharomachrus mocinno* | Resplendent quetzal | Near Threatened | Candidate Bird |
| *Pithecophaga jefferyi* | Philippine Eagle | Critically Endangered | Candidate Bird |
| *Platycercus eximius* | Eastern Rosella | Least Concern | Candidate Bird |
| *Podargus strigoides* | Tawny Frogmouth | Least Concern | Candidate Bird |
| *Poicephalus senegalus* | Senegal Parrot | Least Concern | Candidate Bird |
| *Polemaetus bellicosus* | Martial eagle | Vulnerable | Candidate Bird |
| *Porphyrio hochstetteri* | Takahe | Endangered | Candidate Bird |
| *Probosciger aterrimus* | Palm Cockatoo | Least Concern | Candidate Bird |
| *Psittacula eupatria* | Alexandrine parakeet | Near Threatened | Candidate Bird |
| *Psittacula krameri* | Rose-ringed Parakeet | Least Concern | Candidate Bird |
| *Pyrrhura molinae* | Green-cheeked Parakeet | Least Concern | Candidate Bird |
| *Ramphastos sulfuratus* | Keel-billed Toucan | Least Concern | Candidate Bird |
| *Ramphastos toco* | Toco Toucan | Least Concern | Candidate Bird |
| *Rhea americana* | Greater rhea | Near Threatened | Candidate Bird |
| *Rhodonessa caryophyllacea* | Pink-headed Duck | Critically Endangered | Candidate Bird |
| *Sagittarius serpentarius* | Secretarybird | Vulnerable | Candidate Bird |
| *Sarcogyps calvus* | Red-headed Vulture | Critically Endangered | Candidate Bird |
| *Scolopax minor* | American Woodcock | Least Concern | Candidate Bird |
| *Selasphorus rufus* | Rufous Hummingbird | Least Concern | Candidate Bird |
| *Somateria mollissima* | Common Eider | Near Threatened | Candidate Bird |
| *Sphyrapicus varius* | Yellow-bellied Sapsucker | Least Concern | Candidate Bird |
| *Stephanoaetus coronatus* | Crowned eagle | Near Threatened | Candidate Bird |
| *Streptopelia capicola* | Ring-necked Dove | Least Concern | Candidate Bird |
| *Streptopelia turtur* | European Turtle-dove | Vulnerable | Candidate Bird |
| *Strigops habroptila* | Kakapo | Critically Endangered | Candidate Bird |
| *Strix nebulosa* | Great Grey Owl | Least Concern | Candidate Bird |
| *Strix occidentalis* | Spotted owl | Near Threatened | Candidate Bird |
| *Strix varia* | Barred Owl | Least Concern | Candidate Bird |
| *Surnia ulula* | Northern Hawk-owl | Least Concern | Candidate Bird |
| *Tadorna ferruginea* | Ruddy Shelduck | Least Concern | Candidate Bird |
| *Tauraco erythrolophus* | Red-crested Turaco | Least Concern | Candidate Bird |
| *Terathopius ecaudatus* | Bateleur | Near Threatened | Candidate Bird |
| *Tetrao urogallus* | Western Capercaillie | Least Concern | Candidate Bird |
| *Tympanuchus cupido* | Greater prairie chicken | Vulnerable | Candidate Bird |
| *Tympanuchus phasianellus* | Sharp-tailed Grouse | Least Concern | Candidate Bird |
| *Tyto alba* | Common Barn-owl | Least Concern | Candidate Bird |
| *Upupa epops* | Common Hoopoe | Least Concern | Candidate Bird |
| *Vanellus vanellus* | Northern Lapwing | Near Threatened | Candidate Bird |
| *Zenaida macroura* | Mourning Dove | Least Concern | Candidate Bird |
|  |  |  |  |
| *Acinonyx jubatus* | Cheetah, Hunting Leopard | Vulnerable | Candidate Mammal |
| *Addax nasomaculatus* | Addax | Critically Endangered | Candidate Mammal |
| *Ailuropoda melanoleuca* | Giant Panda | Vulnerable | Candidate Mammal |
| *Ailurops melanotis* | Talaud Bear Cuscus | Critically Endangered | Candidate Mammal |
| *Ailurops ursinus* | Bear Cuscus, Bear Phalanger, Sulawesi Bear Cuscus | Vulnerable | Candidate Mammal |
| *Ailurus fulgens* | Red Panda, Lesser Panda, Red Cat-bear, Tolai Hare | Endangered | Candidate Mammal |
| *Alouatta belzebul* | Red-handed Howler Monkey | Vulnerable | Candidate Mammal |
| *Alouatta discolor* | Spix's Red-handed Howler Monkey | Vulnerable | Candidate Mammal |
| *Alouatta pigra* | Black Howling Monkey | Endangered | Candidate Mammal |
| *Alouatta ululata* | Maranhão Red-handed Howler Monkey | Endangered | Candidate Mammal |
| *Ammotragus lervia* | Aoudad, Barbary Sheep, Uaddan | Vulnerable | Candidate Mammal |
| *Arctictis binturong* | Binturong, Bearcat, Palawan Binturong | Vulnerable | Candidate Mammal |
| *Ateles belzebuth* | White-bellied spider monkey | Endangered | Candidate Mammal |
| *Ateles chamek* | Peruvian spider-monkey | Endangered | Candidate Mammal |
| *Ateles fusciceps* | Black-headed Spider Monkey | Critically Endangered | Candidate Mammal |
| *Ateles geoffroyi* | Geoffroy's spider monkey | Endangered | Candidate Mammal |
| *Ateles hybridus* | White-cheeked spider monkey | Endangered | Candidate Mammal |
| *Ateles marginatus* | Guiana Spider Monkey, Black Spider Monkey, Red-faced Black Spider Monkey | Vulnerable | Candidate Mammal |
| *Ateles paniscus* | Guiana Spider Monkey | Vulnerable | Candidate Mammal |
| *Babyrousa babyrussa* | Hairy Babirusa | Vulnerable | Candidate Mammal |
| *Babyrousa celebensis* | Sulawesi Babirusa | Vulnerable | Candidate Mammal |
| *Babyrousa togeanensis* | Togian Islands Babirusa | Endangered | Candidate Mammal |
| *Bdeogale omnivora* | Sokoko Mongoose | Vulnerable | Candidate Mammal |
| *Blastocerus dichotomus* | Marsh deer | Vulnerable | Candidate Mammal |
| *Bos gaurus* | Gaur | Vulnerable | Candidate Mammal |
| *Bos javanicus* | Banteng | Endangered | Candidate Mammal |
| *Bos mutus* | Wild Yak | Vulnerable | Candidate Mammal |
| *Bos sauveli* | Kouprey | Critically Endangered | Candidate Mammal |
| *Brachyteles arachnoides* | Southern Muriqui | Endangered | Candidate Mammal |
| *Brachyteles hypoxanthus* | Northern Muriqui | Critically Endangered | Candidate Mammal |
| *Bradypus pygmaeus* | Pygmy Three-toed Sloth | Critically Endangered | Candidate Mammal |
| *Bradypus torquatus* | Maned Three-toed Sloth | Vulnerable | Candidate Mammal |
| *Bubalus arnee* | Wild water buffalo | Endangered | Candidate Mammal |
| *Bubalus depressicornis* | Lowland anoa | Endangered | Candidate Mammal |
| *Bubalus mindorensis* | Tamaraw | Critically Endangered | Candidate Mammal |
| *Bubalus quarlesi* | Mountain anoa | Endangered | Candidate Mammal |
| *Budorcas taxicolor* | Takin | Vulnerable | Candidate Mammal |
| *Cacajao calvus* | Bald uakari | Vulnerable | Candidate Mammal |
| *Cacajao hosomi* | Black-headed Uacari | Vulnerable | Candidate Mammal |
| *Camelus ferus* | Bactrian Camel | Critically Endangered | Candidate Mammal |
| *Canis simensis* | Ethiopian wolf | Endangered | Candidate Mammal |
| *Capra walie* | Walia Ibex | Endangered | Candidate Mammal |
| *Cebus flavius* | Blonde Capuchin | Critically Endangered | Candidate Mammal |
| *Cebus kaapori* | Ka’apor Capuchin | Critically Endangered | Candidate Mammal |
| *Cebus robustus* | Crested Capuchin | Endangered | Candidate Mammal |
| *Cebus xanthosternos* | Golden-bellied capuchin | Critically Endangered | Candidate Mammal |
| *Cercocebus atys* | Sooty mangabey | Near Threatened | Candidate Mammal |
| *Cercocebus galeritus* | Tana River Mangabey | Endangered | Candidate Mammal |
| *Cercocebus sanjei* | Sanje mangabey | Endangered | Candidate Mammal |
| *Cercocebus torquatus* | Collared mangabey | Vulnerable | Candidate Mammal |
| *Cercopithecus diana* | Diana monkey | Vulnerable | Candidate Mammal |
| *Cercopithecus dryas* | Dryas Monkey | Critically Endangered | Candidate Mammal |
| *Cercopithecus erythrogaster* | Red-bellied Monkey | Vulnerable | Candidate Mammal |
| *Cercopithecus erythrotis* | Red-eared Monkey | Vulnerable | Candidate Mammal |
| *Cercopithecus hamlyni* | Owl-faced Monkey | Vulnerable | Candidate Mammal |
| *Cercopithecus lhoesti* | L'hoest's monkey | Vulnerable | Candidate Mammal |
| *Cercopithecus preussi* | Preuss’s Monkey | Endangered | Candidate Mammal |
| *Cercopithecus sclateri* | Sclater's Monkey | Vulnerable | Candidate Mammal |
| *Cercopithecus solatus* | Sun-tailed Monkey | Vulnerable | Candidate Mammal |
| *Chiropotes albinasus* | White-nosed Saki | Endangered | Candidate Mammal |
| *Chiropotes satanas* | Black Bearded Saki | Critically Endangered | Candidate Mammal |
| *Chiropotes utahickae* | Uta Hick’s Bearded Saki | Endangered | Candidate Mammal |
| *Chlorocebus djamdjamensis* | Bale Mountains vervet | Vulnerable | Candidate Mammal |
| *Choeropsis liberiensis* | Pygmy Hippopotamus | Endangered | Candidate Mammal |
| *Chrotogale owstoni* | Owston's Civet | Endangered | Candidate Mammal |
| *Colobus polykomos* | King colobus | Vulnerable | Candidate Mammal |
| *Colobus satanas* | Black colobus | Vulnerable | Candidate Mammal |
| *Colobus vellerosus* | White-thighed Colobus | Vulnerable | Candidate Mammal |
| *Cryptoprocta ferox* | Fossa | Vulnerable | Candidate Mammal |
| *Cuon alpinus* | Dhole | Endangered | Candidate Mammal |
| *Cynogale bennettii* | Otter Civet, Otter-civet, Sunda Otter Civet | Endangered | Candidate Mammal |
| *Dendrolagus dorianus* | Doria's Tree Kangaroo | Vulnerable | Candidate Mammal |
| *Dendrolagus goodfellowi* | Goodfellow's tree-kangaroo | Endangered | Candidate Mammal |
| *Dendrolagus inustus* | Grizzled Tree Kangaroo | Vulnerable | Candidate Mammal |
| *Dendrolagus matschiei* | Huon Tree Kangaroo | Endangered | Candidate Mammal |
| *Dendrolagus mayri* | Wondiwoi Tree-kangaroo | Critically Endangered | Candidate Mammal |
| *Dendrolagus mbaiso* | Dingiso | Endangered | Candidate Mammal |
| *Dendrolagus notatus* | Ifola tree-kangaroo | Endangered | Candidate Mammal |
| *Dendrolagus pulcherrimus* | Golden-mantled Tree Kangaroo | Critically Endangered | Candidate Mammal |
| *Dendrolagus scottae* | Tenkile | Critically Endangered | Candidate Mammal |
| *Dendrolagus stellarum* | Seri’s Tree Kangaroo | Vulnerable | Candidate Mammal |
| *Dendrolagus ursinus* | Vogelkop Tree Kangaroo | Vulnerable | Candidate Mammal |
| *Dicerorhinus sumatrensis* | Sumatran Rhinoceros | Critically Endangered | Candidate Mammal |
| *Diceros bicornis* | Black Rhinoceros, Hook-lipped Rhinoceros | Critically Endangered | Candidate Mammal |
| *Diplogale hosei* | Hose's Civet | Vulnerable | Candidate Mammal |
| *Elephas maximus* | Asian Elephant, Indian Elephant | Endangered | Candidate Mammal |
| *Equus africanus* | African Wild Ass | Critically Endangered | Candidate Mammal |
| *Equus ferus* | Przewalski's Horse | Endangered | Candidate Mammal |
| *Equus grevyi* | Grevy's Zebra | Endangered | Candidate Mammal |
| *Equus hemionus* | Asiatic Wild Ass | Near Threatened | Candidate Mammal |
| *Equus zebra* | Mountain Zebra, Hartmann's Mountain Zebra | Vulnerable | Candidate Mammal |
| *Eulemur albifrons* | White-headed lemur | Endangered | Candidate Mammal |
| *Eulemur cinereiceps* | White-collared Lemur | Critically Endangered | Candidate Mammal |
| *Eulemur collaris* | Collared brown lemur | Endangered | Candidate Mammal |
| *Eulemur coronatus* | Crowned Lemur | Endangered | Candidate Mammal |
| *Eulemur macaco* | Black Lemur | Vulnerable | Candidate Mammal |
| *Eulemur mongoz* | Mongoose Lemur | Critically Endangered | Candidate Mammal |
| *Eulemur rubriventer* | Red-bellied Lemur | Vulnerable | Candidate Mammal |
| *Eulemur sanfordi* | Sanford's Brown Lemur | Endangered | Candidate Mammal |
| *Genetta cristata* | Crested Genet | Vulnerable | Candidate Mammal |
| *Genetta johnstoni* | Johnston's Genet | Near Threatened | Candidate Mammal |
| *Gorilla beringei* | Eastern Gorilla | Critically Endangered | Candidate Mammal |
| *Gorilla gorilla* | Lowland Gorilla, Western Gorilla | Critically Endangered | Candidate Mammal |
| *Hapalemur alaotrensis* | Alaotra Reed Lemur | Critically Endangered | Candidate Mammal |
| *Hapalemur aureus* | Golden Bamboo Lemur | Critically Endangered | Candidate Mammal |
| *Hapalemur meridionalis* | Bamboo lemur | Vulnerable | Candidate Mammal |
| *Hapalemur occidentalis* | Sambirano Lesser Bamboo Lemur, Western Gentle Lemur, Western Grey Bamboo Lemur, Western Lesser Bamboo Lemur | Vulnerable | Candidate Mammal |
| *Helarctos malayanus* | Malayan Sun Bear, Sun Bear | Vulnerable | Candidate Mammal |
| *Herpestes fuscus* | Brown Mongoose | Least Concern | Candidate Mammal |
| *Hippopotamus amphibius* | Common Hippopotamus, Hippopotamus, Large Hippo | Vulnerable | Candidate Mammal |
| *Hoolock hoolock* | Western Hoolock Gibbon, Hoolock Gibbon, Western Hoolock | Endangered | Candidate Mammal |
| *Hoolock leuconedys* | Eastern Hoolock Gibbon | Vulnerable | Candidate Mammal |
| *Hylobates agilis* | Agile gibbon | Endangered | Candidate Mammal |
| *Hylobates albibarbis* | Bornean white-bearded gibbon | Endangered | Candidate Mammal |
| *Hylobates klossii* | Kloss’s Gibbon | Endangered | Candidate Mammal |
| *Hylobates lar* | Lar Gibbon | Endangered | Candidate Mammal |
| *Hylobates moloch* | Silvery Gibbon | Endangered | Candidate Mammal |
| *Hylobates muelleri* | Muller's gibbon | Endangered | Candidate Mammal |
| *Hylobates pileatus* | Pileated Gibbon | Endangered | Candidate Mammal |
| *Indri indri* | Indri | Critically Endangered | Candidate Mammal |
| *Kobus megaceros* | Nile Lechwe | Endangered | Candidate Mammal |
| *Lagothrix cana* | Peruvian Woolly Monkey | Endangered | Candidate Mammal |
| *Lagothrix lagotricha* | Brown woolly monkey | Vulnerable | Candidate Mammal |
| *Lagothrix lugens* | Colombian Woolly Monkey | Critically Endangered | Candidate Mammal |
| *Lagothrix poeppigii* | Silvery woolly monkey | Vulnerable | Candidate Mammal |
| *Leontopithecus chrysomelas* | Golden-headed Lion Tamarin | Endangered | Candidate Mammal |
| *Leontopithecus rosalia* | Golden Lion Tamarin | Endangered | Candidate Mammal |
| *Leopardus guigna* | Kodkod | Vulnerable | Candidate Mammal |
| *Leopardus jacobita* | Andean Cat | Endangered | Candidate Mammal |
| *Leopardus tigrinus* | Oncilla | Vulnerable | Candidate Mammal |
| *Liberiictis kuhni* | Liberian Mongoose | Vulnerable | Candidate Mammal |
| *Loxodonta africana* | African elephant | Vulnerable | Candidate Mammal |
| *Lycaon pictus* | African wild dog | Endangered | Candidate Mammal |
| *Macaca arctoides* | Stump-tailed Macaque, Bear Macaque, Stumptail Macaque | Vulnerable | Candidate Mammal |
| *Macaca hecki* | Heck's Macaque | Vulnerable | Candidate Mammal |
| *Macaca leonina* | Northern Pig-tailed Macaque | Vulnerable | Candidate Mammal |
| *Macaca maura* | Moor Macaque | Endangered | Candidate Mammal |
| *Macaca munzala* | Arunachal Macaque | Endangered | Candidate Mammal |
| *Macaca nemestrina* | Southern pig-tailed macaque | Vulnerable | Candidate Mammal |
| *Macaca nigra* | Celebes Crested Macaque | Critically Endangered | Candidate Mammal |
| *Macaca nigrescens* | Gorontalo Macaque | Vulnerable | Candidate Mammal |
| *Macaca ochreata* | Booted Macaque | Vulnerable | Candidate Mammal |
| *Macaca pagensis* | Pagai Island Macaqu | Critically Endangered | Candidate Mammal |
| *Macaca siberu* | Siberut Macaque | Vulnerable | Candidate Mammal |
| *Macaca silenus* | Lion-tailed Macaque | Endangered | Candidate Mammal |
| *Macaca sinica* | Toque Macaque | Endangered | Candidate Mammal |
| *Macaca tonkeana* | Tonkean macaque | Vulnerable | Candidate Mammal |
| *Macrogalidia musschenbroekii* | Brown Palm Civet, Musang, Sulawesi Civet, Sulawesi Palm Civet | Vulnerable | Candidate Mammal |
| *Mandrillus leucophaeus* | Drill | Endangered | Candidate Mammal |
| *Mandrillus sphinx* | Mandrill | Vulnerable | Candidate Mammal |
| *Melursus ursinus* | Sloth Bear | Vulnerable | Candidate Mammal |
| *Muntiacus vuquangensis* | Large-antlered Muntjac | Critically Endangered | Candidate Mammal |
| *Nasalis larvatus* | Proboscis monkey | Endangered | Candidate Mammal |
| *Neofelis diardi* | Sunda coulded leopard | Vulnerable | Candidate Mammal |
| *Neofelis nebulosa* | Clouded Leopard | Vulnerable | Candidate Mammal |
| *Nomascus concolor* | Black Crested Gibbon | Critically Endangered | Candidate Mammal |
| *Nomascus gabriellae* | Red-cheeked Gibbon | Endangered | Candidate Mammal |
| *Nomascus hainanus* | Hainan Gibbon | Critically Endangered | Candidate Mammal |
| *Nomascus leucogenys* | Northern White-cheeked Gibbon | Critically Endangered | Candidate Mammal |
| *Nomascus nasutus* | Cao-vit Gibbon | Critically Endangered | Candidate Mammal |
| *Nomascus siki* | Southern White-cheeked Gibbon | Endangered | Candidate Mammal |
| *Oreonax flavicauda* | Peruvian Yellow-tailed Woolly Monkey | Critically Endangered | Candidate Mammal |
| *Oryx leucoryx* | Arabian Oryx | Vulnerable | Candidate Mammal |
| *Pan paniscus* | Bonobo | Endangered | Candidate Mammal |
| *Pan troglodytes* | Chimpanzee, Common Chimpanzee, Robust Chimpanzee | Endangered | Candidate Mammal |
| *Panthera leo* | Lion | Vulnerable | Candidate Mammal |
| *Panthera tigris* | Tiger | Endangered | Candidate Mammal |
| *Panthera uncia* | Ounce, Snow Leopard | Vulnerable | Candidate Mammal |
| *Pantholops hodgsonii* | Chiru | Near Threatened | Candidate Mammal |
| *Paradoxurus zeylonensis* | Golden Palm Civet | Least Concern | Candidate Mammal |
| *Pardofelis badia* | Borneo Bay Cat, Bay Cat, Bornean Bay Cat, Bornean Marbled Cat | Endangered | Candidate Mammal |
| *Pardofelis marmorata* | Marbled Cat | Near Threatened | Candidate Mammal |
| *Phalanger alexandrae* | Gebe Cuscus | Least Concern | Candidate Mammal |
| *Phalanger lullulae* | Woodlark Cuscus | Least Concern | Candidate Mammal |
| *Phalanger matabiru* | Blue-eyed Cuscus | Vulnerable | Candidate Mammal |
| *Pithecia albicans* | Buffy Saki | Vulnerable | Candidate Mammal |
| *Pongo abelii* | Sumatran Orangutan | Critically Endangered | Candidate Mammal |
| *Pongo pygmaeus* | Bornean Orangutan | Critically Endangered | Candidate Mammal |
| *Porcula salvania* | Pygmy Hog | Critically Endangered | Candidate Mammal |
| *Presbytis chrysomelas* | Sarawak surili | Critically Endangered | Candidate Mammal |
| *Presbytis comata* | Javan Surili | Endangered | Candidate Mammal |
| *Presbytis frontata* | White-fronted surili | Vulnerable | Candidate Mammal |
| *Presbytis hosei* | Hose’s Langur | Vulnerable | Candidate Mammal |
| *Presbytis melalophos* | Sumatran surili | Endangered | Candidate Mammal |
| *Presbytis natunae* | Natuna Island Surili | Vulnerable | Candidate Mammal |
| *Presbytis potenziani* | Mentawai Langur | Endangered | Candidate Mammal |
| *Presbytis thomasi* | Thomas’s Langur | Vulnerable | Candidate Mammal |
| *Priodontes maximus* | Giant Armadillo | Vulnerable | Candidate Mammal |
| *Prionailurus planiceps* | Flat-headed Cat | Endangered | Candidate Mammal |
| *Prionailurus rubiginosus* | Rusty-spotted Cat | Near Threatened | Candidate Mammal |
| *Prionailurus viverrinus* | Fishing Cat | Vulnerable | Candidate Mammal |
| *Procolobus badius* | Western red colobus | Endangered | Candidate Mammal |
| *Procolobus gordonorum* | Udzungwa red colobus | Endangered | Candidate Mammal |
| *Procolobus kirkii* | Zanzibar Red Colobus | Endangered | Candidate Mammal |
| *Procolobus preussi* | Preuss’s Red Colobus | Critically Endangered | Candidate Mammal |
| *Procyon pygmaeus* | Pygmy Raccoon | Critically Endangered | Candidate Mammal |
| *Prolemur simus* | Greater Bamboo Lemu | Critically Endangered | Candidate Mammal |
| *Propithecus candidus* | Silky sifaka | Critically Endangered | Candidate Mammal |
| *Propithecus coquereli* | Coquerel's Sifaka | Endangered | Candidate Mammal |
| *Propithecus coronatus* | Crowned Sifaka | Endangered | Candidate Mammal |
| *Propithecus deckenii* | Van Der Decken's Sifaka, Decken's Sifaka | Endangered | Candidate Mammal |
| *Propithecus diadema* | Diademed Sifaka | Critically Endangered | Candidate Mammal |
| *Propithecus edwardsi* | Milne-Edward's Sifaka | Endangered | Candidate Mammal |
| *Propithecus perrieri* | Perrier’s Sifaka | Critically Endangered | Candidate Mammal |
| *Propithecus tattersalli* | Golden-crowned Sifaka | Critically Endangered | Candidate Mammal |
| *Propithecus verreauxi* | Verreaux's Sifaka | Endangered | Candidate Mammal |
| *Pseudalopex fulvipes* | Darwin's Fox | Endangered | Candidate Mammal |
| *Pseudoryx nghetinhensis* | Saola | Critically Endangered | Candidate Mammal |
| *Pteronura brasiliensis* | Giant otter | Endangered | Candidate Mammal |
| *Pteropus livingstonii* | Livingstone's Flying Fo | Critically Endangered | Candidate Mammal |
| *Pteropus voeltzkowi* | Pemba Flying Fox | Vulnerable | Candidate Mammal |
| *Pygathrix cinerea* | Grey-shanked Douc Langur | Critically Endangered | Candidate Mammal |
| *Pygathrix nemaeus* | Red-shanked Douc Langur | Endangered | Candidate Mammal |
| *Pygathrix nigripes* | Black-shanked Douc Langur | Endangered | Candidate Mammal |
| *Rhinoceros sondaicus* | Javan Rhinoceros | Critically Endangered | Candidate Mammal |
| *Rhinoceros unicornis* | Indian Rhinoceros, Great Indian Rhinoceros | Vulnerable | Candidate Mammal |
| *Rhinopithecus avunculus* | Tonkin Snub-nosed Monkey | Critically Endangered | Candidate Mammal |
| *Rhinopithecus bieti* | Black Snub-nosed Monkey | Endangered | Candidate Mammal |
| *Rhinopithecus brelichi* | Grey Snub-nosed Monkey | Endangered | Candidate Mammal |
| *Rhinopithecus roxellana* | Golden snub-nosed monkey | Endangered | Candidate Mammal |
| *Rucervus eldii* | Eld's Deer | Endangered | Candidate Mammal |
| *Rungwecebus kipunji* | Kipunji | Critically Endangered | Candidate Mammal |
| *Saguinus bicolor* | Brazilian Bare-faced Tamarin | Endangered | Candidate Mammal |
| *Saiga tatarica* | Saiga/mongolian Saiga, Saiga, Saiga Antelope | Critically Endangered | Candidate Mammal |
| *Semnopithecus ajax* | Kashmir Gray Langur | Endangered | Candidate Mammal |
| *Semnopithecus hypoleucos* | Black-footed Gray Langur | Vulnerable | Candidate Mammal |
| *Simias concolor* | Pig-tailed Snub-nosed Langur | Critically Endangered | Candidate Mammal |
| *Spilocuscus papuensis* | Waigeo Cuscus | Vulnerable | Candidate Mammal |
| *Spilocuscus rufoniger* | Black-spotted Cuscus | Critically Endangered | Candidate Mammal |
| *Spilocuscus wilsoni* | Blue-eyed Spotted Cuscus | Critically Endangered | Candidate Mammal |
| *Strigocuscus celebensis* | Small Sulawesi Cuscus, Little Celebes Cuscus, Small Cuscus | Vulnerable | Candidate Mammal |
| *Sus ahoenobarbus* | Palawan Bearded Pig | Near Threatened | Candidate Mammal |
| *Sus barbatus* | Bearded Pig, Western Bearded Pig | Vulnerable | Candidate Mammal |
| *Sus cebifrons* | Visayan Warty Pig | Critically Endangered | Candidate Mammal |
| *Sus oliveri* | Oliver's Warty Pig | Vulnerable | Candidate Mammal |
| *Sus philippensis* | Philippine Warty Pig | Vulnerable | Candidate Mammal |
| *Sus verrucosus* | Javan Warty Pig | Endangered | Candidate Mammal |
| *Symphalangus syndactylus* | Siamang | Endangered | Candidate Mammal |
| *Tapirus bairdii* | Baird's Tapir, Central American Tapir | Endangered | Candidate Mammal |
| *Tapirus indicus* | Asian Tapir, Indian Tapir, Malay Tapir, Malayan Tapir | Endangered | Candidate Mammal |
| *Tapirus pinchaque* | Mountain Tapir, Andean Tapir, Woolly Tapir | Endangered | Candidate Mammal |
| *Tapirus terrestris* | Lowland Tapir, Brazilian Tapir, South American Tapir | Vulnerable | Candidate Mammal |
| *Trachypithecus auratus* | Javan Lutung | Vulnerable | Candidate Mammal |
| *Trachypithecus delacouri* | Delacour's Langur | Critically Endangered | Candidate Mammal |
| *Trachypithecus francoisi* | François’s Langur | Endangered | Candidate Mammal |
| *Trachypithecus geei* | Gee’s Golden Langur | Endangered | Candidate Mammal |
| *Trachypithecus germaini* | Indochinese Lutung | Endangered | Candidate Mammal |
| *Trachypithecus hatinhensis* | Hatinh Langur | Endangered | Candidate Mammal |
| *Trachypithecus johnii* | Nilgiri Langur | Vulnerable | Candidate Mammal |
| *Trachypithecus laotum* | Laotian Langur | Vulnerable | Candidate Mammal |
| *Trachypithecus phayrei* | Phayre’s Leaf-monkey | Endangered | Candidate Mammal |
| *Trachypithecus pileatus* | Capped langur | Vulnerable | Candidate Mammal |
| *Trachypithecus poliocephalus* | Golden-headed Langur | Critically Endangered | Candidate Mammal |
| *Trachypithecus shortridgei* | Shortridge’s Langur | Endangered | Candidate Mammal |
| *Trachypithecus vetulus* | Purple-faced Langur | Endangered | Candidate Mammal |
| *Tragelaphus buxtoni* | Mountain Nyala | Endangered | Candidate Mammal |
| *Tremarctos ornatus* | Andean Bear, Spectacled Bear | Vulnerable | Candidate Mammal |
| *Varecia rubra* | Red Ruffed Lemur, Red-ruffed Lemur | Critically Endangered | Candidate Mammal |
| *Varecia variegata* | Black-and-white Ruffed Lemur, Ruffed Lemur | Critically Endangered | Candidate Mammal |
| *Viverra civettina* | Malabar Civet | Critically Endangered | Candidate Mammal |
| *Viverra megaspila* | Large-spotted Civet | Endangered | Candidate Mammal |
|  |  |  |  |
| *Agkistrodon contortrix* | Northern Copperhead | Least Concern | Candidate Reptile |
| *Agkistrodon piscivorus* | Cottonmouth | Least Concern | Candidate Reptile |
| *Alligator mississippiensis* | American Alligator | Lower Risk/least concern | Candidate Reptile |
| *Alligator sinensis* | Chinese Alligator | Critically Endangered | Candidate Reptile |
| *Anolis carolinensis* | Green Anole | Least Concern | Candidate Reptile |
| *Caiman crocodilus* | Spectacled Caiman | Lower Risk/least concern | Candidate Reptile |
| *Caiman yacare* | Yacaré | Lower Risk/least concern | Candidate Reptile |
| *Chamaeleo calyptratus* | Veiled Chameleon | Least Concern | Candidate Reptile |
| *Chelydra serpentina* | Snapping Turtle | Least Concern | Candidate Reptile |
| *Chlamydosaurus kingii* | Frilled Lizard | Least Concern | Candidate Reptile |
| *Chrysemys picta* | Painted Turtle | Least Concern | Candidate Reptile |
| *Crocodylus acutus* | American crocodile | Vulnerable | Candidate Reptile |
| *Crocodylus intermedius* | Orinoco crocodile | Critically Endangered | Candidate Reptile |
| *Crocodylus johnsoni* | Australian Freshwater Crocodile | Least Concern | Candidate Reptile |
| *Crocodylus niloticus* | Nile Crocodile | Lower Risk/least concern | Candidate Reptile |
| *Crocodylus palustris* | Mugger crocodile | Vulnerable | Candidate Reptile |
| *Crocodylus porosus* | Salt-water Crocodile | Lower Risk/least concern | Candidate Reptile |
| *Crocodylus siamensis* | Siamese crocodile | Critically Endangered | Candidate Reptile |
| *Crotalus adamanteus* | Eastern Diamond-backed Rattlesnake | Least Concern | Candidate Reptile |
| *Crotalus atrox* | Western Diamond-backed Rattlesnake | Least Concern | Candidate Reptile |
| *Crotalus horridus* | Timber Rattlesnake | Least Concern | Candidate Reptile |
| *Dendroaspis polylepis* | Black Mamba | Least Concern | Candidate Reptile |
| *Dermochelys coriacea* | Leatherback | Vulnerable | Candidate Reptile |
| *Emys orbicularis* | European pond turtle | Lower Risk/near threatened | Candidate Reptile |
| *Furcifer pardalis* | Panther Chameleon | Least Concern | Candidate Reptile |
| *Gavialis gangeticus* | Gharial | Critically Endangered | Candidate Reptile |
| *Heloderma suspectum* | Gila monster | Near Threatened | Candidate Reptile |
| *Hierophis viridiflavus* | Western Whip Snake | Least Concern | Candidate Reptile |
| *Lacerta agilis* | Sand Lizard | Least Concern | Candidate Reptile |
| *Lacerta viridis* | Green Lizard | Least Concern | Candidate Reptile |
| *Macrochelys temminckii* | Alligator snapping turtle | Vulnerable | Candidate Reptile |
| *Melanosuchus niger* | Black caiman | Lower Risk/conservation dependent | Candidate Reptile |
| *Morelia viridis* | Green Tree Python | Least Concern | Candidate Reptile |
| *Natrix tessellata* | Tessellated Water Snake | Least Concern | Candidate Reptile |
| *Ophiophagus hannah* | King cobra | Vulnerable | Candidate Reptile |
| *Pelodiscus sinensis* | Chinese softshell turtle | Vulnerable | Candidate Reptile |
| *Podarcis muralis* | Common Wall Lizard | Least Concern | Candidate Reptile |
| *Python bivittatus* | Burmese python | Vulnerable | Candidate Reptile |
| *Python regius* | Royal Python | Least Concern | Candidate Reptile |
| *Thamnophis sirtalis* | Common Gartersnake | Least Concern | Candidate Reptile |
| *Trachemys scripta* | Yellow-bellied Slider Turtle | Least Concern | Candidate Reptile |
| *Varanus salvator* | Common Water Monitor | Least Concern | Candidate Reptile |
| *Vipera ammodytes* | Nose-horned Viper | Least Concern | Candidate Reptile |
| *Vipera aspis* | Asp Viper | Least Concern | Candidate Reptile |
| *Zamenis longissimus* | Aesculapian Ratsnake | Least Concern | Candidate Reptile |
| *Zootoca vivipara* | Viviparous Lizard | Least Concern | Candidate Reptile |
