# Supplementary material for: Conservation prioritization can resolve the flagship species conundrum
Source: Nat Commun. 2020 Feb 24;11:994. doi: 10.1038/s41467-020-14554-z (PMC7040008; doi:10.1038/s41467-020-14554-z)
Supplement: Supplementary file 5 — Supplementary Data Table 2 [file 41467_2020_14554_MOESM5_ESM.docx]

Supplementary Data for

**Conservation prioritization can resolve the flagship species conundrum**

J. McGowan^1,2,3*^, L. J. Beaumont^1^, R. J. Smith^4^, A. L. M. Chauvenet^2,5^, R. Harcourt^1^, S. Atkinson^2^, J. C. Mittermeier^6^, M. Esperon-Rodriguez^1,7^, J. B. Baumgartner^1,8^, A. Beattie^1^, R. Y. Dudaniec^1^, R. Grenyer^6^, D. A. Nipperess^1^, A. Stow^1^, and H. P Possingham^2,3^

**Supplementary Data Table 2. List of flagship species resulting from the integrated approach for scenario h** (Table 2 in main document). For Site IDs, please see Supplementary Table 4. Information is compiled from the IUCN Red List.

| **Site** | **Class** | **Order** | **Family** | **Species** | **Common name** | **IUCN status** | **Current Population Trend** | **Countries of occurrence** |
| --- | --- | --- | --- | --- | --- | --- | --- | --- |
| 1 | Aves | Bucerotiformes | Bucerotidae | *Buceros bicornis* | Great Hornbill | Near Threatened | Decreasing | Bhutan; Cambodia; China; India; Indonesia; Lao People's Democratic Republic; Malaysia; Myanmar; Nepal; Thailand; Viet Nam; Bangladesh |
| 1 | Aves | Bucerotiformes | Bucerotidae | *Buceros rhinoceros* | Rhonocerous hornbill | Near Threatened | Decreasing | Brunei Darussalam; Indonesia; Malaysia; Thailand |
| 1 | Mammalia | Carnivora | Canidae | *Cuon alpinus* | Dhole | Endangered | Decreasing | Bangladesh; Bhutan; Cambodia; China; India; Indonesia; Lao People's Democratic Republic; Malaysia; Myanmar; Nepal; Thailand |
| 1 | Mammalia | Carnivora | Felidae | *Neofelis diardi* | Sunda coulded leopard | Vulnerable | Decreasing | Brunei Darussalam; Indonesia (Kalimantan, Sumatera); Malaysia (Sabah, Sarawak) |
| 1 | Mammalia | Carnivora | Felidae | *Panthera tigris* | Tiger | Endangered | Decreasing | Bangladesh; Bhutan; China (Anhui - Regionally Extinct, Beijing - Regionally Extinct, Chongqing - Regionally Extinct, Fujian - Possibly Extinct, Guangdong - Possibly Extinct, Guangxi - Regionally Extinct, Guizhou - Regionally Extinct, Hebei - Regionally Extinct, Heilongjiang, Henan - Regionally Extinct, Hubei - Regionally Extinct, Hunan - Possibly Extinct, Jiangsu - Regionally Extinct, Jiangxi - Possibly Extinct, Jilin, Liaoning - Regionally Extinct, Shaanxi - Possibly Extinct, Shandong - Regionally Extinct, Shanghai - Regionally Extinct, Shanxi - Regionally Extinct, Sichuan - Regionally Extinct, Tianjin - Regionally Extinct, Tibet [or Xizang], Xinjiang - Regionally Extinct, Yunnan, Zhejiang - Possibly Extinct); India; Indonesia (Bali - Regionally Extinct, Jawa - Regionally Extinct, Sumatera); Lao People's Democratic Republic; Malaysia (Peninsular Malaysia); Myanmar; Nepal; Russian Federation; Thailand |
| 1 | Mammalia | Carnivora | Felidae | *Pardofelis marmorata* | Marbled Cat | Near Threatened | Decreasing | Bangladesh; Bhutan; Brunei Darussalam; Cambodia; China; India; Indonesia (Kalimantan, Sumatera); Lao People's Democratic Republic; Malaysia (Peninsular Malaysia, Sabah, Sarawak); Myanmar; Nepal; Thailand; Viet Nam |
| 1 | Mammalia | Carnivora | Felidae | *Prionailurus planiceps* | Flat-headed Cat | Endangered | Decreasing | Brunei Darussalam; Indonesia (Kalimantan, Sumatera); Malaysia (Peninsular Malaysia, Sabah, Sarawak) |
| 1 | Mammalia | Carnivora | Ursidae | *Helarctos malayanus* | Malayan Sun Bear, Sun Bear | Vulnerable | Decreasing | Bangladesh; Brunei Darussalam; Cambodia; India; Indonesia; Lao People's Democratic Republic; Malaysia; Myanmar; Thailand; Viet Nam |
| 1 | Mammalia | Carnivora | Viverridae | *Arctictis binturong* | Binturong, Bearcat, Palawan Binturong | Vulnerable | Decreasing | Bangladesh; Bhutan; Cambodia; China; India; Indonesia (Jawa, Kalimantan, Sumatera); Lao People's Democratic Republic; Malaysia (Peninsular Malaysia, Sabah, Sarawak); Myanmar; Nepal; Philippines; Thailand; Viet Nam |
| 1 | Mammalia | Carnivora | Viverridae | *Cynogale bennettii* | Otter Civet, Otter-civet, Sunda Otter Civet | Endangered | Decreasing | Brunei Darussalam; Indonesia (Kalimantan, Sumatera); Malaysia (Peninsular Malaysia, Sabah, Sarawak) |
| 1 | Mammalia | Cetartiodactyla | Suidae | *Sus barbatus* | Bearded Pig, Western Bearded Pig | Vulnerable | Decreasing | Brunei Darussalam; Indonesia (Kalimantan, Sumatera); Malaysia (Peninsular Malaysia, Sabah, Sarawak) |
| 1 | Mammalia | Perissodactyla | Tapiridae | *Tapirus indicus* | Asian Tapir, Indian Tapir, Malay Tapir, Malayan Tapir | Endangered | Decreasing | ndonesia (Sumatera); Malaysia; Myanmar; Thailand |
| 1 | Mammalia | Primates | Cercopithecidae | *Macaca nemestrina* | Southern pig-tailed macaque | Vulnerable | Decreasing | Brunei Darussalam; Indonesia (Kalimantan); Malaysia (Peninsular Malaysia, Sabah); Thailand; Singapore |
| 1 | Mammalia | Primates | Cercopithecidae | *Presbytis melalophos* | Sumatran surili | Endangered | Decreasing | Indonesia (Sumatera) |
| 1 | Mammalia | Primates | Hylobatidae | *Hylobates agilis* | Agile gibbon | Endangered | Decreasing | indonesia (Kalimantan, Sumatera); Malaysia (Peninsular Malaysia); Thailand |
| 1 | Mammalia | Primates | Hylobatidae | *Symphalangus syndactylus* | Siamang | Endangered | Decreasing | Indonesia (Sumatera); Malaysia (Peninsular Malaysia); Thailand |
| 1 | Mammalia | Proboscidea | Elephantidae | *Elephas maximus* | Asian Elephant, Indian Elephant | Endangered | Decreasing | Bangladesh; Bhutan; Cambodia; China; India; Indonesia (Kalimantan, Sumatera); Lao People's Democratic Republic; Malaysia (Peninsular Malaysia, Sabah); Myanmar; Nepal; Sri Lanka; Thailand; Viet Nam |
| 1 | Reptilia | Squamata | Elapidae | *Ophiophagus hannah* | King cobra | Vulnerable | Decreasing | Bangladesh; Bhutan; Brunei Darussalam; Cambodia; China; Hong Kong; India (Andaman Is., Andhra Pradesh, Arunachal Pradesh, Assam, Bihar, Goa, Jharkand, Karnataka, Kerala, Manipur, Meghalaya, Mizoram, Nagaland, Orissa, Sikkim, Tamil Nadu, Tripura, Uttaranchal, Uttar Pradesh, West Bengal); Indonesia (Bali, Jawa, Kalimantan, Sulawesi, Sumatera); Lao People's Democratic Republic; Malaysia (Peninsular Malaysia, Sabah, Sarawak); Myanmar; Nepal; Philippines; Singapore; Thailand; Viet Nam |
| 2 | Aves | Accipitriformes | Accipitridae | *Aquila heliaca* | Eastern imperial eagle | Vulnerable | Decreasing | Afghanistan; Armenia; Austria; Azerbaijan; Bangladesh; Bhutan; Bosnia and Herzegovina; Bulgaria; Cambodia; China; Croatia; Czech Republic; Djibouti; Egypt; Eritrea; Ethiopia; Georgia; Greece; Hong Kong; Belarus; Cameroon; Cyprus; Denmark; Finland; France; Germany; Italy; Libya; Lithuania; Malaysia; Morocco; Poland; Singapore; Slovenia; Sweden; Togo Hungary; India; Iran, Islamic Republic of; Iraq; Israel; Japan; Jordan; Kazakhstan; Kenya; Korea, Democratic People's Republic of; Korea, Republic of; Kuwait; Kyrgyzstan; Lao People's Democratic Republic; Lebanon; Macao; Macedonia, the former Yugoslav Republic of; Moldova; Mongolia; Montenegro; Myanmar; Nepal; Oman; Pakistan; Palestinian Territory, Occupied; Qatar; Romania; Russian Federation (Central Asian Russia, Eastern Asian Russia, European Russia); Saudi Arabia; Serbia; Slovakia; Sudan; Syrian Arab Republic; Taiwan, Province of China; Tajikistan; Tanzania, United Republic of; Thailand; Turkey; Turkmenistan; Ukraine; United Arab Emirates; Uzbekistan; Viet Nam; Yemen |
| 2 | Aves | Accipitriformes | Accipitridae | *Gypaetus barbatus* | Bearded vulture | Near Threatened | Decreasing | Afghanistan; Algeria; Andorra; Armenia; Azerbaijan; Bhutan; China; Egypt; Eritrea; Ethiopia; France; Georgia; Greece; India; Iran, Islamic Republic of; Iraq; Israel; Kazakhstan; Kenya; Kyrgyzstan; Lesotho; Mongolia; Morocco; Nepal; Pakistan; Russian Federation (Central Asian Russia, Eastern Asian Russia, European Russia); Saudi Arabia; South Africa; Spain; Sudan; Tajikistan; Tanzania, United Republic of; Turkey; Turkmenistan; Uganda; Uzbekistan; Yemen; Austria; Italy; Switzerland; Bulgaria; Croatia; Cyprus; Czech Republic; Djibouti; Germany; Korea, Democratic People's Republic of; Lebanon; Mauritania; Mozambique; Namibia; Portugal; Romania; Somalia; Zimbabwe |
| 2 | Aves | Accipitriformes | Accipitridae | *Gyps africanus* | White backed vulture | Critically Endangered | Decreasing | Angola; Benin; Botswana; Burkina Faso; Burundi; Cameroon; Central African Republic; Chad; Congo, The Democratic Republic of the; Côte d'Ivoire; Eritrea; Ethiopia; Gambia; Ghana; Guinea; Guinea-Bissau; Kenya; Malawi; Mali; Mauritania; Mozambique; Namibia; Niger; Nigeria; Rwanda; Senegal; Sierra Leone; Somalia; South Africa; South Sudan; Sudan; Swaziland; Tanzania, United Republic of; Togo; Uganda; Zambia; Zimbabwe; Lesotho; Liberia |
| 2 | Aves | Accipitriformes | Accipitridae | *Neophron percnopterus* | Egyptian Vulture | Endangered | Decreasing | Afghanistan; Albania; Algeria; Andorra; Angola; Armenia; Azerbaijan; Benin; Bulgaria; Burkina Faso; Cameroon; Cape Verde; Central African Republic; Chad; Cyprus; Djibouti; Egypt; Eritrea; Ethiopia; France; Georgia; Ghana; Gibraltar; Greece; Guinea; India; Iran, Islamic Republic of; Iraq; Israel; Italy; Jordan; Kazakhstan; Kenya; Kuwait; Kyrgyzstan; Lebanon; Libya; Macedonia, the former Yugoslav Republic of; Mali; Malta; Mauritania; Morocco; Namibia; Nepal; Niger; Nigeria; Oman; Pakistan; Palestinian Territory, Occupied; Portugal; Russian Federation (Central Asian Russia - Vagrant, European Russia); Saudi Arabia; Senegal; Somalia; South Sudan; Spain (Canary Is.); Sudan; Syrian Arab Republic; Tajikistan; Tanzania, United Republic of; Togo; Tunisia; Turkey; Turkmenistan; United Arab Emirates; Uzbekistan; Western Sahara; Yemen; Austria; Bangladesh; Belgium; Botswana; Congo, The Democratic Republic of the; Czech Republic; Denmark; Estonia; Finland; Gambia; Hungary; Mongolia; Mozambique; Myanmar; Norway; Poland; Qatar; Slovakia; Slovenia; Sri Lanka; Svalbard and Jan Mayen; Sweden; Switzerland; United Kingdom; Zimbabwe; China; Côte d'Ivoire; Guinea-Bissau; Uganda |
| 2 | Aves | Accipitriformes | Accipitridae | *Polemaetus bellicosus* | Martial eagle | Vulnerable | Decreasing | Angola; Benin; Botswana; Burkina Faso; Burundi; Cameroon; Central African Republic; Chad; Congo, The Democratic Republic of the; Côte d'Ivoire; Eritrea; Ethiopia; Gambia; Ghana; Guinea; Guinea-Bissau; Kenya; Malawi; Mali; Mauritania; Mozambique; Namibia; Niger; Nigeria; Rwanda; Senegal; Sierra Leone; Somalia; South Africa; South Sudan; Sudan; Swaziland; Tanzania, United Republic of; Togo; Uganda; Zambia; Zimbabwe; Liberia |
| 2 | Aves | Accipitriformes | Accipitridae | *Terathopius ecaudatus* | Bateleur | Near Threatened | Decreasing | Angola; Benin; Botswana; Burkina Faso; Burundi; Cameroon; Central African Republic; Chad; Congo; Congo, The Democratic Republic of the; Côte d'Ivoire; Djibouti; Egypt; Eritrea; Ethiopia; Gabon; Gambia; Ghana; Guinea; Guinea-Bissau; Kenya; Malawi; Mali; Mauritania; Mozambique; Namibia; Niger; Nigeria; Rwanda; Saudi Arabia; Senegal; Sierra Leone; Somalia; South Africa; South Sudan; Sudan; Swaziland; Tanzania, United Republic of; Togo; Uganda; Yemen; Zambia; Zimbabwe; Iraq; Israel; Lesotho; Liberia; Tunisia |
| 2 | Aves | Accipitriformes | Sagittariidae | *Sagittarius serpentarius* | Secretarybird | Vulnerable | Decreasing | Angola; Benin; Botswana; Burkina Faso; Burundi; Cameroon; Central African Republic; Chad; Congo, The Democratic Republic of the; Côte d'Ivoire; Djibouti; Eritrea; Ethiopia; Ghana; Kenya; Lesotho; Malawi; Mali; Mauritania; Mozambique; Namibia; Niger; Nigeria; Senegal; Somalia; South Africa; South Sudan; Sudan; Swaziland; Tanzania, United Republic of; Togo; Uganda; Zambia; Zimbabwe; Liberia; Rwanda; Guinea-Bissau |
| 2 | Aves | Gruiformes | Gruidae | *Balearica regulorum* | Grey Crowned-crane | Endangered | Decreasing | Angola; Botswana; Burundi; Congo, The Democratic Republic of the; Kenya; Malawi; Mozambique; Namibia; Rwanda; South Africa; Tanzania, United Republic of; Uganda; Zambia; Zimbabwe; Lesotho; Swaziland |
| 2 | Aves | Otidiformes | Otididae | *Ardeotis kori* | Kori bustard | Near Threatened | Decreasing | Angola; Botswana; Ethiopia; Kenya; Mozambique; Namibia; Somalia; South Africa; South Sudan; Sudan; Tanzania, United Republic of; Uganda; Zambia; Zimbabwe |
| 2 | Aves | Psittaciformes | Psittacidae | *Agapornis fischeri* | Fischer's lovebird | Near Threatened | Decreasing | Tanzania, United Republic of; Burundi; Kenya; Rwanda; Uganda |
| 2 | Mammalia | Carnivora | Felidae | *Acinonyx jubatus* | Cheetah, Hunting Leopard | Vulnerable | Decreasing | Algeria; Angola; Benin; Botswana; Burkina Faso; Central African Republic; Chad; Ethiopia; Iran, Islamic Republic of; Kenya; Mali; Mozambique; Namibia; Niger; South Africa; South Sudan; Tanzania, United Republic of; Uganda; Zambia; Zimbabwe; Swaziland |
| 2 | Mammalia | Cetartiodactyla | Hippopotamidae | *Hippopotamus amphibius* | Common Hippopotamus, Hippopotamus, Large Hippo | Vulnerable | Stable | Angola; Benin; Botswana; Burkina Faso; Burundi; Cameroon; Central African Republic; Chad; Congo; Congo, The Democratic Republic of the; Côte d'Ivoire; Equatorial Guinea; Ethiopia; Gabon; Gambia; Ghana; Guinea; Guinea-Bissau; Kenya; Malawi; Mali; Mozambique; Namibia; Niger; Nigeria; Rwanda; Senegal; Sierra Leone; Somalia; South Africa; South Sudan; Sudan; Swaziland; Tanzania, United Republic of; Togo; Uganda; Zambia; Zimbabwe |
| 2 | Mammalia | Perissodactyla | Rhinocerotidae | *Diceros bicornis* | Black Rhinoceros, Hook-lipped Rhinoceros | Critically Endangered | Increasing | Angola; Kenya; Mozambique; Namibia; South Africa; Tanzania, United Republic of; Zimbabwe; Botswana; Malawi; Swaziland; Zambia |
| 2 | Mammalia | Proboscidea | Elephantidae | *Loxodonta africana* | African elephant | Vulnerable | Increasing | Angola; Benin; Botswana; Burkina Faso; Cameroon; Central African Republic; Chad; Congo; Congo, The Democratic Republic of the; Côte d'Ivoire; Equatorial Guinea; Eritrea; Ethiopia; Gabon; Ghana; Guinea; Guinea-Bissau; Kenya; Liberia; Malawi; Mali; Mozambique; Namibia; Niger; Nigeria; Rwanda; Senegal; Sierra Leone; Somalia; South Africa; South Sudan; Tanzania, United Republic of; Togo; Uganda; Zambia; Zimbabwe; Swaziland |
| 3 | Aves | Accipitriformes | Accipitridae | *Harpia harpyja* | Harpy eagle | Near Threatened | Decreasing | Argentina; Belize; Bolivia, Plurinational States of; Brazil; Colombia; Costa Rica; Ecuador; French Guiana; Guatemala; Guyana; Honduras; Mexico; Nicaragua; Panama; Paraguay; Peru; Suriname; Venezuela, Bolivarian Republic of |
| 3 | Aves | Caprimulgiformes | Apodidae | *Chaetura pelagica* | Chimney swift | Near Threatened | Decreasing | Aruba; Bahamas; Belize; Bermuda; Brazil; Canada; Cayman Islands; Chile; Colombia; Costa Rica; Cuba; Dominican Republic; Ecuador; El Salvador; Guatemala; Haiti; Honduras; Mexico; Nicaragua; Panama; Peru; Puerto Rico; Saint Pierre and Miquelon; Turks and Caicos Islands; United States; Venezuela, Bolivarian Republic of; Anguilla; Barbados; Greenland; Jamaica; Portugal; United Kingdom; Virgin Islands, U.S. |
| 3 | Mammalia | Carnivora | Felidae | *Leopardus tigrinus* | Oncilla | Vulnerable | Decreasing | Bolivia, Plurinational States of; Brazil; Colombia; Costa Rica; Ecuador; French Guiana; Guyana; Panama; Peru; Suriname; Venezuela, Bolivarian Republic of |
| 3 | Mammalia | Carnivora | Mustelidae | *Pteronura brasiliensis* | Giant otter | Endangered | Decreasing | Bolivia, Plurinational States of; Brazil; Colombia; Ecuador; French Guiana; Guyana; Paraguay; Peru; Suriname; Venezuela, Bolivarian Republic of |
| 3 | Mammalia | Cingulata | Chlamyphoridae | *Priodontes maximus* | Giant Armadillo | Vulnerable | Decreasing | Argentina (Chaco, Formosa, Salta, Santiago del Estero); Bolivia, Plurinational States of; Brazil (Acre, Amapá, Amazonas, Espírito Santo, Goiás, Maranhão, Mato Grosso, Mato Grosso do Sul, Minas Gerais, Pará, Paraná, Rondônia, Roraima, Tocantins); Colombia (Colombia (mainland)); Ecuador (Ecuador (mainland)); French Guiana; Guyana; Paraguay; Peru; Suriname; Venezuela, Bolivarian Republic of (Venezuela (mainland)) |
| 3 | Mammalia | Perissodactyla | Tapiridae | *Tapirus terrestris* | Lowland Tapir, Brazilian Tapir, South American Tapir | Vulnerable | Decreasing | Argentina; Bolivia, Plurinational States of; Brazil; Colombia; Ecuador; French Guiana; Guyana; Paraguay; Peru; Suriname; Venezuela, Bolivarian Republic of |
| 3 | Mammalia | Primates | Atelidae | *Ateles belzebuth* | White-bellied spider monkey | Endangered | Decreasing | Brazil (Amazonas, Roraima); Colombia (Colombia (mainland)); Ecuador (Ecuador (mainland)); Peru; Venezuela, Bolivarian Republic of |
| 3 | Mammalia | Primates | Atelidae | *Ateles chamek* | Peruvian spider-monkey | Endangered | Decreasing | Bolivia, Plurinational States of; Brazil (Acre, Amazonas, Mato Grosso, Rondônia); Peru |
| 3 | Mammalia | Primates | Atelidae | *Lagothrix lagotricha* | Brown woolly monkey | Vulnerable | Decreasing | Brazil (Amazonas); Colombia (Colombia (mainland)); Ecuador (Ecuador (mainland)); Peru |
| 3 | Mammalia | Primates | Atelidae | *Lagothrix poeppigii* | Silvery woolly monkey | Vulnerable | Decreasing | Brazil (Acre, Amazonas); Ecuador (Ecuador (mainland)); Peru |
| 3 | Mammalia | Primates | Pitheciidae | *Cacajao calvus* | Bald uakari | Vulnerable | Decreasing | Brazil; Peru |
| 3 | Reptilia | Crocodylia | Alligatoridae | *Melanosuchus niger* | Black caiman | Lower Risk/conservation dependent | Need updating | Bolivia, Plurinational States of; Brazil; Colombia; Ecuador; French Guiana; Guyana; Peru |
| 4 | Aves | Accipitriformes | Accipitridae | *Stephanoaetus coronatus* | Crowned eagle | Near Threatened | Decreasing | Angola; Burundi; Cameroon; Central African Republic; Congo; Congo, The Democratic Republic of the; Côte d'Ivoire; Equatorial Guinea; Ethiopia; Gabon; Ghana; Guinea; Guinea-Bissau; Kenya; Liberia; Malawi; Mozambique; Nigeria; Rwanda; Senegal; Sierra Leone; South Africa; South Sudan; Sudan; Swaziland; Tanzania, United Republic of; Togo; Uganda; Zambia; Zimbabwe; Benin; Botswana |
| 4 | Mammalia | Carnivora | Herpestidae | *Liberiictis kuhni* | Liberian Mongoose | Vulnerable | Decreasing | Côte d'Ivoire; Liberia |
| 4 | Mammalia | Carnivora | Viverridae | *Genetta johnstoni* | Johnston's Genet | Near Threatened | Decreasing | Côte d'Ivoire; Ghana; Guinea; Liberia; Senegal; Sierra Leone |
| 4 | Mammalia | Cetartiodactyla | Hippopotamidae | *Choeropsis liberiensis* | Pygmy Hippopotamus | Endangered | Decreasing | Côte d'Ivoire; Guinea; Liberia; Sierra Leone |
| 4 | Mammalia | Primates | Cercopithecidae | *Cercocebus atys* | Sooty mangabey | Near Threatened | Decreasing | Côte d'Ivoire; Guinea; Guinea-Bissau; Liberia; Senegal; Sierra Leone |
| 4 | Mammalia | Primates | Cercopithecidae | *Cercopithecus diana* | Diana monkey | Vulnerable | Decreasing | Côte d'Ivoire; Guinea; Liberia; Sierra Leone |
| 4 | Mammalia | Primates | Cercopithecidae | *Colobus polykomos* | King colobus | Vulnerable | Unknown | Côte d'Ivoire; Guinea; Guinea-Bissau; Liberia; Sierra Leone |
| 4 | Mammalia | Primates | Cercopithecidae | *Procolobus badius* | Western red colobus | Endangered | Decreasing | Côte d'Ivoire; Guinea; Liberia; Sierra Leone |
| 4 | Mammalia | Primates | Hominidae | *Pan troglodytes* | Chimpanzee, Common Chimpanzee, Robust Chimpanzee | Endangered | Decreasing | Angola; Burundi; Cameroon; Central African Republic; Congo; Congo, The Democratic Republic of the; Côte d'Ivoire; Equatorial Guinea (Equatorial Guinea (mainland)); Gabon; Ghana; Guinea; Guinea-Bissau; Liberia; Mali; Nigeria; Rwanda; Senegal; Sierra Leone; South Sudan; Tanzania, United Republic of; Uganda |
| 5 | Mammalia | Carnivora | Ursidae | *Tremarctos ornatus* | Andean Bear, Spectacled Bear | Vulnerable | Decreasing | Bolivia, Plurinational States of; Colombia; Ecuador; Peru; Venezuela, Bolivarian Republic of |
| 5 | Mammalia | Perissodactyla | Tapiridae | *Tapirus pinchaque* | Mountain Tapir, Andean Tapir, Woolly Tapir | Endangered | Decreasing | Colombia; Ecuador; Peru |
| 5 | Mammalia | Primates | Atelidae | *Ateles fusciceps* | Black-headed Spider Monkey | Critically Endangered | Decreasing | Colombia (Colombia (mainland)); Ecuador (Ecuador (mainland)); Panama |
| 5 | Reptilia | Crocodylia | Crocodylidae | *Crocodylus acutus* | American crocodile | Vulnerable | Increasing | Belize; Colombia; Costa Rica; Cuba; Dominican Republic; Ecuador; El Salvador; Guatemala; Haiti; Honduras; Jamaica; Mexico; Nicaragua; Panama; Peru; United States (Florida); Venezuela, Bolivarian Republic of |
| 6 | Aves | Accipitriformes | Accipitridae | *Aegypius monachus* | Cinereous vulture | Near Threatened | Decreasing | Afghanistan; Armenia; Azerbaijan; Bhutan; Bulgaria; China; Croatia; Georgia; Greece; India; Iran, Islamic Republic of; Iraq; Israel; Kazakhstan; Korea, Democratic People's Republic of; Korea, Republic of; Kuwait; Kyrgyzstan; Lebanon; Macedonia, the former Yugoslav Republic of; Mongolia; Montenegro; Myanmar; Nepal; Pakistan; Portugal; Russian Federation (Central Asian Russia, Eastern Asian Russia, European Russia); Saudi Arabia; Serbia; Spain; Sudan; Syrian Arab Republic; Tajikistan; Turkey; Turkmenistan; Ukraine; Uzbekistan; Viet Nam; France; Austria; Bangladesh; Belarus; Bosnia and Herzegovina; Cambodia; Egypt; Germany; Gibraltar; Hungary; Japan; Jordan; Latvia; Malaysia; Netherlands; Oman; Poland; Slovakia; Switzerland; Taiwan, Province of China; Thailand; Tunisia; Yemen; Albania; Morocco |
| 6 | Aves | Accipitriformes | Accipitridae | *Gyps bengalensis* | White-rumped vulture | Critically Endangered | Decreasing | Afghanistan; Bangladesh; Bhutan; Cambodia; India; Iran, Islamic Republic of; Myanmar; Nepal; Pakistan; Brunei Darussalam; Russian Federation (European Russia) |
| 6 | Aves | Accipitriformes | Accipitridae | *Sarcogyps calvus* | Red-headed Vulture | Critically Endangered | Decreasing | Bangladesh; Cambodia; China; India; Lao People's Democratic Republic; Myanmar; Nepal; Viet Nam; Pakistan |
| 6 | Aves | Galliformes | Phasianidae | *Coturnix japonica* | Japanese quail | Near Threatened | Decreasing | Bhutan; China; India; Japan; Korea, Democratic People's Republic of; Korea, Republic of; Lao People's Democratic Republic; Mongolia; Myanmar; Russian Federation (Eastern Asian Russia); Thailand; Viet Nam; taly; Réunion; United States (Hawaiian Is.); Cambodia; Philippines |
| 6 | Aves | Psittaciformes | Psittacidae | *Psittacula eupatria* | Alexandrine parakeet | Near Threatened | Decreasing | Afghanistan; Bangladesh; Bhutan; Cambodia; India; Lao People's Democratic Republic; Myanmar; Nepal; Pakistan; Sri Lanka; Thailand; Viet Nam; Iran, Islamic Republic of; Qatar; Turkey; United Arab Emirates |
| 6 | Mammalia | Carnivora | Ailuridae | *Ailurus fulgens* | Red Panda, Lesser Panda, Red Cat-bear, Tolai Hare | Endangered | Decreasing | Bhutan; China; India; Myanmar; Nepal |
| 6 | Mammalia | Carnivora | Felidae | *Neofelis nebulosa* | Clouded Leopard | Vulnerable | Decreasing | Bangladesh; Bhutan; Cambodia; China; India; Lao People's Democratic Republic; Malaysia (Peninsular Malaysia); Myanmar; Nepal; Thailand; Viet Nam |
| 6 | Mammalia | Carnivora | Felidae | *Prionailurus viverrinus* | Fishing Cat | Vulnerable | Decreasing | Bangladesh; Cambodia; India; Myanmar; Nepal; Pakistan; Sri Lanka; Thailand |
| 6 | Mammalia | Carnivora | Ursidae | *Melursus ursinus* | Sloth Bear | Vulnerable | Decreasing | India; Nepal; Sri Lanka |
| 6 | Mammalia | Cetartiodactyla | Bovidae | *Bos gaurus* | Gaur | Vulnerable | Decreasing | Bhutan; Cambodia; China; India; Lao People's Democratic Republic; Malaysia (Peninsular Malaysia); Myanmar; Nepal; Thailand; Viet Nam |
| 6 | Mammalia | Cetartiodactyla | Bovidae | *Bubalus arnee* | Wild water buffalo | Endangered | Decreasing | Bhutan; Cambodia; India; Myanmar; Nepal; Thailand |
| 6 | Mammalia | Cetartiodactyla | Suidae | *Porcula salvania* | Pygmy Hog | Critically Endangered | Decreasing | India |
| 6 | Mammalia | Perissodactyla | Rhinocerotidae | *Rhinoceros unicornis* | Indian Rhinoceros, Great Indian Rhinoceros | Vulnerable | Increasing | India; Nepal |
| 6 | Mammalia | Primates | Cercopithecidae | *Macaca arctoides* | Stump-tailed Macaque, Bear Macaque, Stumptail Macaque | Vulnerable | Decreasing | Cambodia; China; India; Lao People's Democratic Republic; Malaysia; Myanmar; Thailand; Viet Nam; Hong Kong |
| 6 | Mammalia | Primates | Cercopithecidae | *Macaca leonina* | Macaca leonina | Vulnerable | Decreasing | Bangladesh; Cambodia; China; India; Lao People's Democratic Republic; Myanmar; Thailand; Viet Nam |
| 6 | Mammalia | Primates | Cercopithecidae | *Trachypithecus pileatus* | Capped langur | Vulnerable | Decreasing | Bangladesh; Bhutan; India (Assam, Manipur, Meghalaya, Nagaland); Myanmar |
| 6 | Mammalia | Primates | Hylobatidae | *Hoolock hoolock* | Western Hoolock Gibbon, Hoolock Gibbon, Western Hoolock | Endangered | Decreasing | Bangladesh; India (Assam); Myanmar |
| 6 | Reptilia | Crocodylia | Crocodylidae | *Crocodylus palustris* | Mugger crocodile | Vulnerable | Stable | India; Iran, Islamic Republic of; Nepal; Pakistan; Sri Lanka |
| 6 | Reptilia | Crocodylia | Gavialidae | *Gavialis gangeticus* | Gharial | Critically Endangered | Decreasing | ndia (Bihar, Uttar Pradesh); Nepal |
| 6 | Reptilia | Squamata | Pythonidae | *Python bivittatus* | Burmese python | Vulnerable | Decreasing | Bangladesh; Cambodia; China (Fujian, Guangdong, Guangxi, Hainan, Sichuan, Yunnan); Hong Kong; India (Arunachal Pradesh); Indonesia (Bali, Jawa, Sulawesi); Lao People's Democratic Republic; Myanmar; Nepal; Thailand; Viet Nam; Singapore; United States (Florida) |
| 7 | Mammalia | Carnivora | Eupleridae | *Cryptoprocta ferox* | Fossa | Vulnerable | Decreasing | Madagascar |
| 7 | Mammalia | Primates | Indriidae | *Indri indri* | Indri | Critically Endangered | Decreasing | Madagascar |
| 7 | Mammalia | Primates | Indriidae | *Propithecus candidus* | Silky sifaka | Critically Endangered | Decreasing | Madagascar |
| 7 | Mammalia | Primates | Lemuridae | *Eulemur albifrons* | White-headed lemur | Endangered | Decreasing | Madagascar |
| 7 | Mammalia | Primates | Lemuridae | *Eulemur macaco* | Black Lemur | Vulnerable | Decreasing | Madagascar |
| 7 | Mammalia | Primates | Lemuridae | *Eulemur rubriventer* | Red-bellied Lemur | Vulnerable | Decreasing | Madagascar |
| 7 | Mammalia | Primates | Lemuridae | *Hapalemur occidentalis* | Sambirano Lesser Bamboo Lemur, Western Gentle Lemur, Western Grey Bamboo Lemur, Western Lesser Bamboo Lemur | Vulnerable | Decreasing | Madagascar |
| 7 | Mammalia | Primates | Lemuridae | *Varecia rubra* | Red Ruffed Lemur, Red-ruffed Lemur | Critically Endangered | Decreasing | Madagascar |
| 7 | Mammalia | Primates | Lemuridae | *Varecia variegata* | Black-and-white Ruffed Lemur, Ruffed Lemur | Critically Endangered | Decreasing | Madagascar |
| 8 | Aves | Trogoniformes | Trogonidae | *Pharomachrus mocinno* | Resplendent quetzal | Near Threatened | Decreasing | Costa Rica; El Salvador; Guatemala; Honduras; Mexico; Nicaragua; Panama |
| 8 | Mammalia | Perissodactyla | Tapiridae | *Tapirus bairdii* | Baird's Tapir, Central American Tapir | Endangered | Decreasing | Belize; Colombia; Costa Rica; Guatemala; Honduras; Mexico; Nicaragua; Panama |
| 8 | Mammalia | Primates | Atelidae | *Alouatta pigra* | Black Howling Monkey | Endangered | Decreasing | Belize; Guatemala; Mexico (Campeche, Chiapas, Quintana Roo, Tabasco, Yucatán) |
| 8 | Mammalia | Primates | Atelidae | *Ateles geoffroyi* | Geoffroy's spider monkey | Endangered | Decreasing | Belize; Colombia (Colombia (mainland)); Costa Rica (Costa Rica (mainland)); El Salvador; Guatemala; Honduras (Honduras (mainland)); Mexico; Nicaragua (Nicaragua (mainland)); Panama |
| 9 | Aves | Struthioniformes | Casuariidae | *Casuarius bennetti* | Dwarf cassowary | Least Concern | Stable | Indonesia (Papua); Papua New Guinea |
| 9 | Aves | Struthioniformes | Casuariidae | *Casuarius casuarius* | Southern Cassowary | Least Concern | Decreasing | Australia; Indonesia; Papua New Guinea |
| 9 | Mammalia | Diprotodontia | Macropodidae | *Dendrolagus goodfellowi* | Goodfellow's tree-kangaroo | Endangered | Decreasing | Indonesia (Papua); Papua New Guinea |
| 9 | Mammalia | Diprotodontia | Macropodidae | *Dendrolagus notatus* | Ifola tree-kangaroo | Endangered | Decreasing | Papua New Guinea |
| 9 | Mammalia | Diprotodontia | Phalangeridae | *Spilocuscus rufoniger* | Black-spotted Cuscus | Critically Endangered | Decreasing | Indonesia; Papua New Guine |
| 10 | Aves | Psittaciformes | Psittacidae | *Ara militaris* | Military macaw | Vulnerable | Decreasing | Argentina; Bolivia, Plurinational States of; Colombia; Ecuador; Mexico; Peru; Venezuela, Bolivarian Republic of |
| 11 | Mammalia | Carnivora | Felidae | *Panthera leo* | Lion | Vulnerable | Decreasing | Angola; Benin; Botswana; Burkina Faso; Cameroon; Central African Republic; Chad; Congo, The Democratic Republic of the; Ethiopia; India; Kenya; Malawi; Mozambique; Namibia; Niger; Nigeria; Senegal; Somalia; South Africa; South Sudan; Sudan; Swaziland; Tanzania, United Republic of; Uganda; Zambia; Zimbabwe |
| 11 | Mammalia | Carnivora | Herpestidae | *Bdeogale omnivora* | Sokoko Mongoose | Vulnerable | Decreasing | Kenya; Tanzania, United Republic of |
| 12 | Aves | Struthioniformes | Rheidae | *Rhea americana* | Greater rhea | Near Threatened | Decreasing | Argentina; Bolivia, Plurinational States of; Brazil; Paraguay; Uruguay |
| 12 | Mammalia | Cetartiodactyla | Cervidae | *Blastocerus dichotomus* | Marsh deer | Vulnerable | Decreasing | Argentina; Bolivia, Plurinational States of; Brazil; Paraguay; Peru |
| 13 | Mammalia | Primates | Cercopithecidae | *Cercocebus torquatus* | Collared mangabey | Vulnerable | Decreasing | Cameroon; Equatorial Guinea; Gabon; Nigeria |
| 13 | Mammalia | Primates | Cercopithecidae | *Colobus satanas* | Black colobus | Vulnerable | Decreasing | Cameroon; Congo; Equatorial Guinea (Bioko); Gabon |
| 13 | Mammalia | Primates | Cercopithecidae | *Mandrillus sphinx* | Mandrill | Vulnerable | Unknown | Cameroon; Congo; Equatorial Guinea; Gabon |
| 13 | Mammalia | Primates | Hominidae | *Gorilla gorilla* | Lowland Gorilla, Western Gorilla | Critically Endangered | Decreasing | Angola (Cabinda); Cameroon; Central African Republic; Congo; Equatorial Guinea (Equatorial Guinea (mainland)); Gabon; Nigeria |
| 14 | Aves | Piciformes | Picidae | *Leuconotopicus borealis* | Red-cockaded woodpecker | Near Threatened | Decreasing | United States |
| 14 | Aves | Piciformes | Picidae | *Melanerpes erythrocephalus* | Red-headed woodpecker | Near Threatened | Decreasing | Canada; Mexico; United States |
| 15 | Aves | Strigiformes | Strigidae | *Strix occidentalis* | Spotted owl | Near Threatened | Decreasing | Canada; Mexico; United States |
| 15 | Reptilia | Squamata | Helodermatidae | *Heloderma suspectum* | Gila monster | Near Threatened | Decreasing | Mexico; United States |
| 16 | Mammalia | Carnivora | Felidae | *Panthera uncia* | Ounce, Snow Leopard | Vulnerable | Decreasing | Afghanistan; Bhutan; China (Gansu, Nei Mongol, Qinghai, Sichuan, Tibet [or Xizang], Xinjiang, Yunnan); India (Arunachal Pradesh, Himachal Pradesh, Jammu-Kashmir, Sikkim, Uttaranchal); Kazakhstan; Kyrgyzstan; Mongolia; Nepal; Pakistan; Russian Federation; Tajikistan; Uzbekistan |
| 16 | Mammalia | Carnivora | Ursidae | *Ailuropoda melanoleuca* | Giant Panda | Vulnerable | Increasing | China (Gansu, Hubei - Regionally Extinct, Hunan - Regionally Extinct, Shaanxi, Sichuan) |
| 16 | Mammalia | Cetartiodactyla | Bovidae | *Budorcas taxicolor* | Takin | Vulnerable | Decreasing | Bhutan; China; India; Myanmar |
| 16 | Mammalia | Primates | Cercopithecidae | *Rhinopithecus roxellana* | Golden snub-nosed monkey | Endangered | Decreasing | China (Gansu, Hubei, Shanxi, Sichuan) |
| 16 | Reptilia | Testudines | Trionychidae | *Pelodiscus sinensis* | Chinese softshell turtle | Vulnerable | Decreasing | China; Japan; Taiwan, Province of China; Viet Nam; Thailand; United States |
| 17 | Aves | Accipitriformes | Accipitridae | *Pithecophaga jefferyi* | Philippine Eagle | Critically Endangered | Decreasing | Philippines |
| 17 | Aves | Columbiformes | Columbidae | *Caloenas nicobarica* | Nicobar pigeon | Near Threatened | Decreasing | Cambodia; India; Indonesia; Malaysia; Myanmar; Palau; Papua New Guinea; Philippines; Solomon Islands; Thailand; Viet Nam |
| 17 | Mammalia | Cetartiodactyla | Suidae | *Sus philippensis* | Philippine Warty Pig | Vulnerable | Decreasing | Philippines |
| 18 | Mammalia | Primates | Indriidae | *Propithecus verreauxi* | Verreaux's Sifaka | Endangered | Decreasing | Madagascar |
| 18 | Mammalia | Primates | Lemuridae | *Eulemur collaris* | Collared brown lemur | Endangered | Decreasing | Madagascar |
| 18 | Mammalia | Primates | Lemuridae | *Hapalemur meridionalis* | Bamboo lemur | Vulnerable | Decreasing | Madagascar |
| 20 | Mammalia | Carnivora | Felidae | *Pardofelis badia* | Borneo Bay Cat, Bay Cat, Bornean Bay Cat, Bornean Marbled Cat | Endangered | Decreasing | Indonesia (Kalimantan); Malaysia (Sabah, Sarawak) |
| 20 | Mammalia | Primates | Cercopithecidae | *Nasalis larvatus* | Proboscis monkey | Endangered | Decreasing | Brunei Darussalam; Indonesia (Kalimantan); Malaysia (Sabah, Sarawak) |
| 20 | Mammalia | Primates | Cercopithecidae | *Presbytis chrysomelas* | Sarawak surili | Critically Endangered | Decreasing | Brunei Darussalam; Indonesia (Kalimantan); Malaysia (Sarawak) |
| 20 | Mammalia | Primates | Cercopithecidae | *Presbytis frontata* | White-fronted surili | Vulnerable | Decreasing | Indonesia (Kalimantan); Malaysia (Sarawak) |
| 20 | Mammalia | Primates | Hominidae | *Pongo pygmaeus* | Bornean Orangutan | Critically Endangered | Decreasing | Indonesia (Kalimantan); Malaysia (Sabah, Sarawak) |
| 20 | Mammalia | Primates | Hylobatidae | *Hylobates muelleri* | Muller's gibbon | Endangered | Decreasing | Indonesia (Kalimantan) |
| 20 | Reptilia | Crocodylia | Crocodylidae | *Crocodylus siamensis* | Siamese crocodile | Critically Endangered | Decreasing | Cambodia; Indonesia (Jawa - Possibly Extinct, Kalimantan); Lao People's Democratic Republic; Thailand; Viet Nam |
| 25 | Aves | Gruiformes | Gruidae | *Anthropoides paradiseus* | Blue crane | Vulnerable | Stable | Namibia; South Africa; Botswana; Lesotho; Swaziland; Zimbabwe |
| 26 | Aves | Psittaciformes | Cacatuidae | *Cacatua sulphurea* | Yellow-crested cockatoo | Critically Endangered | Decreasing | Indonesia; Timor-Leste; Singapore |
| 26 | Mammalia | Carnivora | Viverridae | *Macrogalidia musschenbroekii* | Brown Palm Civet, Musang, Sulawesi Civet, Sulawesi Palm Civet | Vulnerable | Decreasing | Indonesia (Sulawesi) |
| 26 | Mammalia | Cetartiodactyla | Bovidae | *Bubalus depressicornis* | Lowland anoa | Endangered | Decreasing | Indonesia (Sulawesi) |
| 26 | Mammalia | Cetartiodactyla | Bovidae | *Bubalus quarlesi* | Mountain anoa | Endangered | Decreasing | Indonesia (Sulawesi) |
| 26 | Mammalia | Cetartiodactyla | Suidae | *Babyrousa celebensis* | Sulawesi Babirusa | Vulnerable | Decreasing | Indonesia |
| 26 | Mammalia | Diprotodontia | Phalangeridae | *Ailurops ursinus* | Bear Cuscus, Bear Phalanger, Sulawesi Bear Cuscus | Vulnerable | Decreasing | Indonesia |
| 26 | Mammalia | Diprotodontia | Phalangeridae | *Strigocuscus celebensis* | Small Sulawesi Cuscus, Little Celebes Cuscus, Small Cuscus | Vulnerable | Decreasing | Indonesia |
| 26 | Mammalia | Primates | Cercopithecidae | *Macaca tonkeana* | Tonkean macaque | Vulnerable | Decreasing | Indonesia (Sulawesi) |
| 28 | Aves | Accipitriformes | Accipitridae | *Milvus milvus* | Red kite | Near Threatened | Decreasing | Albania; Andorra; Austria; Belarus; Belgium; Bosnia and Herzegovina; Bulgaria; Croatia; Czech Republic; Denmark; France; Germany; Gibraltar; Hungary; Iran, Islamic Republic of; Italy; Latvia; Liechtenstein; Lithuania; Luxembourg; Malta; Moldova; Morocco; Netherlands; Poland; Portugal; Romania; Russian Federation (European Russia); Serbia; Slovakia; Slovenia; Spain (Canary Is. - Possibly Extinct); Sweden; Switzerland; Tunisia; Turkey; Ukraine; United Kingdom; Armenia; Azerbaijan; Bangladesh; Cyprus; Estonia; Finland; Georgia; Iceland; India; Iraq; Ireland; Israel; Jordan; Lebanon; Libya; Macedonia, the former Yugoslav Republic of; Mauritania; Montenegro; Nepal; Norway; Sudan; Syrian Arab Republic; San Marino |
| 28 | Reptilia | Testudines | Emydidae | *Emys orbicularis* | European pond turtle | Lower Risk/near threatened | Unknown | Albania; Algeria; Armenia; Austria; Azerbaijan; Belarus; Bosnia and Herzegovina; Bulgaria; Croatia; Czech Republic; France; Georgia; Germany; Greece; Hungary; Iran, Islamic Republic of; Italy; Kazakhstan; Latvia; Liechtenstein; Lithuania; Macedonia, the former Yugoslav Republic of; Malta; Moldova; Monaco; Montenegro; Morocco; Netherlands; Poland; Portugal; Romania; Russian Federation; Serbia; Slovakia; Slovenia; Spain; Switzerland; Syrian Arab Republic; Tunisia; Turkey; Turkmenistan; Ukraine; Belgium; Luxembourg; United Kingdom |
| 34 | Aves | Psittaciformes | Psittacidae | *Anodorhynchus hyacinthinus* | Hyacinth macaw | Vulnerable | Decreasing | Bolivia, Plurinational States of; Brazil; Paraguay |
| 34 | Mammalia | Primates | Atelidae | *Ateles marginatus* | White-cheeked spider monkey | Endangered | Decreasing | Brazil (Mato Grosso, Pará) |
| 34 | Mammalia | Primates | Atelidae | *Ateles paniscus* | Guiana Spider Monkey, Black Spider Monkey, Red-faced Black Spider Monkey | Vulnerable | Decreasing | Brazil (Amapá, Pará, Roraima); French Guiana; Guyana; Suriname |
| 35 | Mammalia | Primates | Cercopithecidae | *Cercopithecus lhoesti* | L'hoest's monkey | Vulnerable | Decreasing | Burundi; Congo, The Democratic Republic of the; Rwanda; Uganda |
| 43 | Mammalia | Carnivora | Canidae | *Lycaon pictus* | African wild dog | Endangered | Decreasing | Angola; Benin; Botswana; Burkina Faso; Central African Republic; Chad; Ethiopia; Kenya; Malawi; Mozambique; Namibia; Niger; Senegal; South Africa; South Sudan; Sudan; Tanzania, United Republic of; Zambia; Zimbabwe |
| 52 | Mammalia | Carnivora | Canidae | *Canis simensis* | Ethiopian wolf | Endangered | Decreasing | Ethiopia |
| 52 | Mammalia | Cetartiodactyla | Bovidae | *Tragelaphus buxtoni* | Mountain Nyala | Endangered | Decreasing | Ethiopia |
| 52 | Mammalia | Primates | Cercopithecidae | *Chlorocebus djamdjamensis* | Bale Mountains vervet | Vulnerable | Decreasing | Ethiopia |
| 63 | Mammalia | Cetartiodactyla | Bovidae | *Ammotragus lervia* | Aoudad, Barbary Sheep, Uaddan | Vulnerable | Decreasing | Algeria; Chad; Egypt; Libya; Mali; Mauritania; Morocco; Niger; Sudan; Tunisia; Mexico; Spain (Canary Is.); United States |
| 82 | Mammalia | Carnivora | Felidae | *Leopardus guigna* | Kodkod | Vulnerable | Decreasing | Argentina; Chile |
| 95 | Mammalia | Primates | Cebidae | *Cebus xanthosternos* | Golden-bellied capuchin | Critically Endangered | Decreasing | Brazil (Bahia) |
| 101 | Reptilia | Crocodylia | Crocodylidae | *Crocodylus intermedius* | Orinoco crocodile | Critically Endangered | Decreasing | Colombia; Venezuela, Bolivarian Republic of |
| 102 | Aves | Otidiformes | Otididae | *Otis tarda* | Great Bustard | Vulnerable | Decreasing | Afghanistan; Armenia; Austria; Bulgaria; China; Croatia; Czech Republic; Georgia; Germany; Greece; Hungary; Iran, Islamic Republic of; Iraq; Italy; Kazakhstan; Kyrgyzstan; Macedonia, the former Yugoslav Republic of; Moldova; Mongolia; Montenegro; Morocco; Portugal; Romania; Russian Federation (Central Asian Russia, Eastern Asian Russia, European Russia); Serbia; Slovakia; Spain; Syrian Arab Republic; Tajikistan; Turkey; Turkmenistan; Ukraine; Uzbekistan; Albania; Belgium; Cyprus; Denmark; Egypt; Finland; France; Gibraltar; Ireland; Israel; Japan; Korea, Democratic People's Republic of; Korea, Republic of; Latvia; Lebanon; Luxembourg; Malta; Netherlands; Pakistan; Saudi Arabia; Tunisia |
| 106 | Mammalia | Primates | Indriidae | *Propithecus deckenii* | Van Der Decken's Sifaka, Decken's Sifaka | Endangered | Decreasing | Madagascar |
| 112 | Aves | Galliformes | Phasianidae | *Tympanuchus cupido* | Greater prairie chicken | Vulnerable | Decreasing | United States |
| 112 | Aves | Piciformes | Picidae | *Campephilus principalis* | Ivory-billed woodpecker | Critically Endangered | Decreasing | Cuba; United State |
| 112 | Reptilia | Testudines | Chelydridae | *Macrochelys temminckii* | Alligator snapping turtle | Vulnerable | Need updating | United States |
| 124 | Mammalia | Primates | Cercopithecidae | *Cercocebus sanjei* | Sanje mangabey | Endangered | Decreasing | Tanzania, United Republic of |
| 124 | Mammalia | Primates | Cercopithecidae | *Procolobus gordonorum* | Udzungwa red colobus | Endangered | Decreasing | Tanzania, United Republic of |
| 129 | Aves | Accipitriformes | Accipitridae | *Haliaeetus pelagicus* | Steller's Sea-eagle | Vulnerable | Decreasing | China; Japan; Korea, Democratic People's Republic of; Korea, Republic of; Russian Federation (Eastern Asian Russia); Taiwan, Province of China; United States |
| 129 | Aves | Anseriformes | Anatidae | *Clangula hyemalis* | Long-tailed Duck | Vulnerable | Decreasing | Austria; Azerbaijan; Belarus; Belgium; Bulgaria; Canada; China; Czech Republic; Denmark; Estonia; Faroe Islands; Finland; France; Germany; Greece; Greenland; Hungary; Iceland; India; Iran, Islamic Republic of; Ireland; Italy; Japan; Kazakhstan; Korea, Democratic People's Republic of; Korea, Republic of; Kyrgyzstan; Latvia; Lithuania; Macedonia, the former Yugoslav Republic of; Mexico; Montenegro; Nepal; Netherlands; Norway; Pakistan; Poland; Romania; Russian Federation (Central Asian Russia, Eastern Asian Russia, European Russia); Saint Pierre and Miquelon; Serbia; Slovakia; Slovenia; Spain; Svalbard and Jan Mayen; Sweden; Switzerland; Turkmenistan; Ukraine; United Kingdom; United States; Armenia; Bermuda; Bosnia and Herzegovina; Croatia; Israel; Jordan; Luxembourg; Portugal; Turkey |
| 129 | Aves | Gruiformes | Gruidae | *Grus japonensis* | Red-crowned Crane | Endangered | Decreasing | China; Japan; Korea, Democratic People's Republic of; Korea, Republic of; Mongolia; Russian Federation (Eastern Asian Russia); Taiwan, Province of China |
| 129 | Aves | Strigiformes | Strigidae | *Bubo blakistoni* | Blakiston'sfish owl | Endangered | Decreasing | China; Japan; Russian Federation (Eastern Asian Russia) |
| 130 | Aves | Charadriiformes | Charadriidae | *Charadrius melodus* | Piping plover | Near Threatened | Increasing | Bahamas; Barbados; Bermuda; Canada; Cuba; Dominican Republic; Guadeloupe; Haiti; Jamaica; Martinique; Mexico; Nicaragua; Puerto Rico; Saint Kitts and Nevis; Saint Pierre and Miquelon; Turks and Caicos Islands; United States; Virgin Islands, British; Virgin Islands, U.S.; Anguilla; Antigua and Barbuda; Ecuador; Saint Vincent and the Grenadines |
| 131 | Aves | Psittaciformes | Psittacidae | *Ara glaucogularis* | Blue-throated macaw | Critically Endangered | Stable | Bolivia, Plurinational States of |
| 137 | Aves | Galliformes | Phasianidae | *Centrocercus urophasianus* | Greater sage-grouse | Near Threatened | Decreasing | Canada; United States |
| 145 | Aves | Psittaciformes | Cacatuidae | *Cacatua moluccensis* | Salmon-crested Cockatoo | Vulnerable | Decreasing | Indonesia |
| 146 | Mammalia | Perissodactyla | Equidae | *Equus zebra* | Mountain Zebra, Hartmann's Mountain Zebra | Vulnerable | Unknown | Namibia; South Africa (Eastern Cape Province, Northern Cape Province, Western Cape) |
| 152 | Aves | Gruiformes | Rallidae | *Gallirallus australis* | Weka | Vulnerable | Decreasing | New Zealand |
| 152 | Aves | Gruiformes | Rallidae | *Porphyrio hochstetteri* | Takahe | Endangered | Stable | New Zealand |
| 152 | Aves | Psittaciformes | Strigopidae | *Nestor notabilis* | Kea | Endangered | Decreasing | New Zealand |
| 152 | Aves | Psittaciformes | Strigopidae | *Strigops habroptila* | Kakapo | Critically Endangered | Increasing | New Zealand |
| 157 | Aves | Psittaciformes | Psittacidae | *Amazona oratrix* | Yellow-headed amazon | Endangered | Decreasing | Belize; Guatemala; Mexico |
| 159 | Aves | Charadriiformes | Scolopacidae | *Numenius borealis* | Eskimo Curlew | Critically Endangered | Unknown | Argentina; Barbados; Brazil; Canada; Chile; Mexico; Paraguay; United States; Uruguay |
| 161 | Aves | Struthioniformes | Casuariidae | *Casuarius unappendiculatus* | Northern Cassowary | Least Concern | Decreasing | Indonesia; Papua New Guinea |
| 161 | Mammalia | Diprotodontia | Macropodidae | *Dendrolagus inustus* | Grizzled Tree Kangaroo | Vulnerable | Decreasing | Indonesia; Papua New Guinea |
| 403 | Aves | Gruiformes | Gruidae | *Grus americana* | Whooping Crane | Endangered | Increasing | Canada; United States |
| 527 | Mammalia | Cetartiodactyla | Bovidae | *Saiga tatarica* | Saiga/mongolian Saiga, Saiga, Saiga Antelope | Critically Endangered | Decreasing | Kazakhstan; Mongolia; Russian Federation; Turkmenistan; Uzbekistan |
| 589 | Mammalia | Primates | Hylobatidae | *Hylobates albibarbis* | Bornean white-bearded gibbon | Endangered | Decreasing | Indonesia (Kalimantan) |
