# Supplementary material for: Conservation prioritization can resolve the flagship species conundrum
Source: Nat Commun. 2020 Feb 24;11:994. doi: 10.1038/s41467-020-14554-z (PMC7040008; doi:10.1038/s41467-020-14554-z)
Supplement: Supplementary file 6 — Source Code [file 41467_2020_14554_MOESM6_ESM.pdf]

Source Code for

Conservation prioritization can resolve the flagship species conundrum

J. McGowan[1,2,3*], L. J. Beaumont[1], R. J. Smith[4], A. L. M. Chauvenet[2,5], R. Harcourt[1], S. Atkinson[2], J. C. Mittermeier[6], M. Esperon-Rodriguez[1,7], J. B. Baumgartner[1,8], A. Beattie[1], R. Y. Dudaniec[1], R. Grenyer[6], D. A. Nipperess[1], A. Stow[1], and H. P Possingham[2,3]

1. Department of Biological Sciences, Macquarie University, NSW, 2109, Australia.
2. Centre for Biodiversity and Conservation Science, University of Queensland, QLD,4072, Australia
3. The Nature Conservancy, Arlington, VA, USA
4. Durrell Institute of Conservation and Ecology, School of Anthropology and Conservation, University of Kent, Canterbury, Kent CT2 7NR, UK
5. Environmental Futures Research Institute & School of Environment and Science, Griffith University, QLD, 4222, Australia.
6. School of Geography and Environment, Oxford University, South Parks Road, Oxford, OX1 3QY, UK.
7. Hawkesbury Institute for the Environment, Western Sydney University, 2753 NSW Australia
8. Centre of Excellence for Biosecurity Risk Analysis (CEBRA), School of BioSciences, University of Melbourne, Parkville, Victoria, Australia

Corresponding author: Jennifer McGowan, The Nature Conservancy, Arlington, Virginia, USA; email: jennifer.mcgowan@tnc.org

R programming source code
Code for Integrated Analysis
Written by A. Chauvenet and J. McGowan

The following code assumes you have two matrices organized by the binary presence of species (rows) in each unique site or planning unit (columns, or “PUID” in code). For our analysis, these matrices are coded as “Backtot_m” and “Candtot_m”, which relates to the Background species and Candidate flagship matrices, respectively.

The PU_layer contains attributes that are desirable for breaking ties. You can refer to Supplementary Figure 1 for the schematic of the algorithm below.

```
##Start here by reading in data
PU<-read.csv('PU_Layer.csv',header=T)
Candtot_m<-read.csv("candidatematrix.csv",row=1)
Backtot_m<-read.csv("backgroundmatrix.csv",row=1)

master_back<-Backtot_m #makes duplicate backup
master_cand<-Candtot_m #makes duplicate backup

#### Below is the integrated algorithm from Supplementary Figure 1.

SAVED <- c() ## store the name of the Candidate species selected
SAVED_count <- c()
SAVED2 <-
  c() ##Number of unique new candidates species selected in the Max step (including 0 if no
new species)
PUIDs <- c() ## the puid of the Max
BACK <- c() ## the total number of Background save in each puid
ECO_rem_rec <-
  c() ## the Number of puids removed with each ecoregions

Backtot_m <- master_back # reloads to start fresh each time
Candtot_m <- master_cand

count <- 0 # counting the steps and selection of PUIDS
while (dim(Backtot_m)[1] > 0 &&
       dim(Backtot_m)[2] > 0)
  # condition to keep loop running until nothing left
{
  count <- count + 1

  #1/ find PUID which maximises the number of background species (and candidate species if
there is a tie)
  max_sum <-
    apply(Backtot_m, 2, sum) #sums over each column in the matrix
  len1 <-ifelse(length(max_sum)<100,1,round(1 * length(max_sum) / 100,0))
     #grabs the top 1 percent with max species
  max_N <- sort(max_sum, decreasing = TRUE)[1:len1]
  max_pos <-
```

```
    order(max_sum, decreasing = T)
# position in Backtot_m that has the largest # of background species
  temp <- c()
  for (j in 1:len1) {
    pos2 <-
      which(colnames(Candtot_m) == names(max_sum[max_pos])[j])
##finds the corresponding Number of candidates in the top 1% that is selected
    temp <- c(temp, sum(Candtot_m[, pos2]))
  }
  #breaking ties between candidate sites
  temp_max_pos <- which(temp == max(temp))
  temp_max<-temp[temp_max_pos]
  if (length(temp_max) > 1)
    temp_max_pos <-
    temp_max_pos[which(max_N[temp_max_pos] == max(max_N[temp_max_pos]))]
# if tie, pick the site with the max background species
# above line is where one can assign how to break ties with different attributes, or
# species in this section
 (max_N[temp_max]==max(max_N[temp_max]
  if (length(temp_max_pos) > 1)
    temp_max_pos <-
    sample(temp_max_pos, 1)

# if the max background tie is another tie, break tie at random

  back_puid_max <- max_N[temp_max_pos] #collect and store the selected site
  max_pos <-
    which(colnames(Backtot_m) == names(max_N)[temp_max_pos]) # find the position
  max_puid <-
    colnames(Backtot_m)[max_pos] #find the unique id of selected site

##The following section deals with complementarity by removing the selected site,
#removing its associated ecoregion, and removing all associated species from the background
#species matrix and storing the candidates that have been selected

# storing candidates
  cand_pos <- which(colnames(Candtot_m) == max_puid)
  saved_pos <- which(Candtot_m[, cand_pos] == 1)
  SAVED <-
    c(SAVED, rownames(Candtot_m)[saved_pos]) # storing the candidates from the selected
#site
  diff <- length(SAVED) - length(unique(SAVED))
# if duplicates, find them and only store unique candidates in this list
  SAVED <- unique(SAVED)
  SAVED2 <- c(SAVED2, length(saved_pos) - diff)
  SAVED_count <- c(SAVED_count, rep(count, (length(saved_pos) - diff)))
  print(c("count", count))
  print(c("unique number of candidate saved", sum(SAVED2)))
  PUIDs <- c(PUIDs, max_puid)
```

```
  if (SAVED2[length(SAVED2)] > 0) {
    # removing the site puid after selection from Background matrix

print(c("dim backtot 0", dim(Backtot_m)))
    BACK <- c(BACK, sum(Backtot_m[, max_pos]))
    saved_back <- which(Backtot_m[, max_pos] == 1)
    Backtot_m <- Backtot_m[, -max_pos]
    print(c("dim backtot 1 (rem col)", dim(Backtot_m)))
    if (length(saved_back) > 0)
      Backtot_m <- Backtot_m[-saved_back, ]
    print(c("dim backtot 2 (rem rows)", dim(Backtot_m)))
    ECO_rem_rec <- c(ECO_rem_rec, length(max_pos))

    # removing the associated puids with the ecoregion that has been selected
    ECO_rem <- PU$G200_ID[which(PU$puid == PUIDs[length(PUIDs)])]
    puid_rem <-
      PU$puid[which(PU$G200_ID == ECO_rem)] # all the PUIDs belonging to that
#ecoregion
    temp_puid <- c()
    for (k in 1:length(puid_rem)) {
      temp_puid <- c(temp_puid, which(colnames(Backtot_m) == puid_rem[k]))
    }
    ECO_rem_rec <- c(ECO_rem_rec, length(temp_puid))
    if (length(temp_puid) > 0)
      Backtot_m <- Backtot_m[, -temp_puid]
    print(c("dim backtot 3 (rem col)", dim(Backtot_m)))
  } else{
    Backtot_m <- Backtot_m[, -max_pos]
    BACK <- c(BACK, 0)
    ECO_rem_rec <- c(ECO_rem_rec, 0)
  }

}

#Below helps link stored lists and writes the output files
cbind(SAVED,SAVED_count)

Puids<-as.data.frame(PUIDs)
Back<-as.data.frame(BACK)
Pus<-cbind(Puids,Back)
Pus$search_no<-seq.int(nrow(Pus))
Cands<-cbind(SAVED,SAVED_count)
colnames(Cands)[2]<-"search_no"

Base_result<-merge(Cands,Pus, by="search_no") #final output
write.csv(Base_result,"Base_results_TH.csv",row.names=T)
```

#End Code
